# Neutrophil to Lymphocyte ratio as a predictor for immune-related adverse events in cancer patients treated with immune checkpoint inhibitors: a systematic review and meta-analysis

**DOI:** 10.3389/fimmu.2023.1234142

**Published:** 2023-08-09

**Authors:** Wei Zhang, Yifei Tan, Yuquan Li, Jiang Liu

**Affiliations:** ^1^ Breast Tumor Center, Sun Yat-sen Memorial Hospital, Sun Yat-sen University, Guangzhou, China; ^2^ Department of Ultrasonography, West China Second University Hospital, Sichuan University, Chengdu, China; ^3^ Department of Thoracic Surgery, Sun Yat-sen Memorial Hospital, Sun Yat-Sen University, Guangzhou, China

**Keywords:** immune checkpoint inhibitors, immune-related adverse events, neutrophil to lymphocyte ratio, biomarker, cancer treatment

## Abstract

**Background:**

The use of immune checkpoint inhibitors (ICIs) in cancer treatment has led to an increase in immune-related adverse events (irAEs), which can cause treatment discontinuation and even fatal reactions. The purpose of this study was to evaluate the usefulness of the peripheral biomarker neutrophil to lymphocyte ratio (NLR) in predicting irAEs.

**Methods:**

A systematic search of databases was conducted to identify studies on the predictive value of NLR for irAEs. The standardized mean difference (SMD) was used to compare continuous NLR, while crude odds ratios (ORs) were calculated for categorized NLR if adjusted ORs and 95% confidence intervals (CIs) were not provided in the original study.

**Results:**

The meta-analysis included 47 studies with a total of 11,491 cancer patients treated with ICIs. The baseline continuous NLR was significantly lower in patients with irAEs compared to those without (SMD=-1.55, 95%CI=-2.64 to -0.46, P=0.006). Similarly, categorized NLR showed that lower baseline NLR was associated with increased irAEs (OR=0.55, 95%CI=0.41-0.73, P<0.001). Subgroup analysis revealed that the OR for predicting irAEs with NLR cut-off values of 3 and 5 was 0.4 and 0.59, respectively. Interestingly, increased baseline NLR was associated with a higher incidence of immune-related liver injury (OR=2.44, 95%CI=1.23-4.84, I2 = 0%, P=0.010).

**Conclusion:**

Our study suggests that lower baseline NLR is associated with a higher risk of overall irAEs. However, further studies are needed to determine the best cut-off value and explore the efficacy of NLR in predicting specific types of irAEs.

## Introduction

Over the past few decades, immunotherapies have emerged as a milestone in the treatment of cancer, resulting in durable responses for several types of cancer and extended overall survival (1). Immune checkpoint inhibitors (ICIs) are increasingly being used for certain cancers and have shown remarkable efficacy in promoting long-term survival in patients with metastatic disease. Also, they are gradually becoming a therapeutic option for earlier-stage cancers (2, 3). Currently, the most commonly used ICIs for tumors are anti-programmed death-1 (PD-1) and its ligand (PD-L1) and cytotoxic T-lymphocyte-associated antigen-4 (CTLA-4) inhibitors (4). When tumors occur, PD-L1 expressed on tumor cells binds with PD-1 to down-regulate the response of T cells, allowing tumor cells to escape immune recognition and destruction, thereby promoting tumor growth (5). CTLA-4 is another receptor expressed on T cells that binds to CD80 and CD86 ligands on antigen-presenting cells. This interaction results in weakened activation of effector T cells and participates in tumor immune escape (6).

However, the increased use of ICIs has led to an increase in the occurrence of immune-related adverse events (irAEs) (7). Unlike conventional radiotherapy and chemotherapy, the anticancer immune responses may also result in adverse side effects due to self-tolerance impairment caused by autoreactive lymphocytes and autoantibodies, disruption of normal tissue immune homeostasis, and subsequent off-target immune and inflammatory responses (8). IrAEs can affect any organ or system in the body, most commonly in the skin, gastrointestinal tract, lungs, musculoskeletal system, and endocrine organs such as the thyroid, adrenal gland, and pituitary gland (9). Although most irAEs are mild and manageable when promptly recognized and appropriately treated, some severe irAEs may necessitate the termination of immunotherapy or the addition of immunosuppressants. Moreover, severe or fatal toxic reactions can occur, posing significant challenges to immunotherapy (10, 11). For example, the reported case fatality rate of immune-related myocarditis is approximately 20%-50% (10). Furthermore, once patients are diagnosed with neurotoxicity following ICIs, almost 80% of them are judged to have grade 3-4 neurotoxicity, and approximately one-third of them die due to irAEs (12).

Exploring biomarkers for predicting the efficacy of ICIs has been one of the focuses of immunotherapy (13). Peripheral blood biomarkers are economical, convenient, and easily obtainable in clinical practice, making them a commonly adopted option. Some studies have demonstrated their prognostic value in both therapeutic efficacy and survival outcomes (14). However, due to the possibilities of drug discontinuation or even death caused by irAEs, recent studies have begun to seek predictive indicators for irAEs to prevent the occurrence of side effects earlier (15). The neutrophil-to-lymphocyte ratio (NLR) is an indicator that reflects systematic inflammation (16). Previous studies have demonstrated that an elevated NLR is a significant risk factor associated with poorer survival outcomes in oncological patients, including those diagnosed with lung cancer, breast cancer and hepatocellular carcinoma (17–19). However, the role of NLR in predicting irAEs remains controversial (14, 20). Therefore, the aim of this review and meta-analysis is to evaluate the overall predictive value of NLR in irAEs in patients undergoing immunotherapy and to explore a suitable NLR cutoff for clinical use.

## Materials and methods

This study was designed based on the preferred reporting items for systematic review and meta-analysis (PRISMA) 2020 guidelines (21). The aim was to evaluate the predictive value of peripheral NLR for irAEs in oncological patients treated with ICIs.

### Search strategy

The authors conducted a systematic search of PubMed, Ovid Medline, Embase, and Cochrane Database of Systematic Reviews up to March 25^th^, 2023. Additionally, grey literature was searched using Google Scholar and related conference websites such as the European Society of Medical Oncology and American Society of Clinical Oncology. The search terms used were “immune checkpoint inhibitor,” “immune-related adverse event,” and “neutrophil to lymphocyte ratio.” The detailed search strategy is provided in **Supplementary Table 1**. All the studies containing titles and abstracts were imported into Endnote X9 to find duplicate studies and then for literature screening.

### Selection criteria

Studies were included if they met the following criteria: 1) included cancer patients treated with ICIs, 2) reported the incidence of irAEs, and 3) evaluated NLR as a predictive value for irAE. Exclusion criteria were: 1) *in vitro* or *in vivo* studies, 2) no available data on continuous NLR, categorized number of NLR by cut-off, or odds ratio associated with irAE, and 3) case reports or case series with a sample size of less than 10. There was no restriction on study design, but studies were limited to the English language. Conference could be included if the data could be extracted from the abstracts and other review and meta-analysis were screened for further including studies. Data from the same project or center will be selected as one for further meta-analysis.

### Literature screening, data extraction and quality assessment

Two researchers (W Zhang and YF Tan) independently screened the titles and abstracts according to the inclusion and exclusion criteria. The full text was further evaluated if the abstracts could not be determined or data could not be extracted. Disagreements were resolved by discussion with a third investigator (J Liu). Data from eligible studies were extracted into a standard form that included study characteristics, NLR-related items, patient characteristics, and the incidence of irAE in all or different subtypes. NLR could be recorded in baseline or post-treatment of ICIs. The continuous or categorized number of NLR were collected in terms of adverse event (AE) group and non-AE group. Odd ratios (ORs) with corresponding 95% confidence intervals (CIs) were also collected when available. Multivariate or adjusted ORs were preferentially included, otherwise univariate ORs was included or calculated based on the original data of the article. The quality of included studies was assessed using the Newcastle-Ottawa Scale (NOS) tool (22). Studies with NOS scores higher than 6 were considered of high quality, while studies with NOS scores of 5 or less were defined as moderate quality.

### Statistical analysis

The main outcome of the meta-analysis was the predictive value of NLR for irAE in cancer patients. If the continuous NLR provided as medians and ranges instead of means and standard deviations (SD), the authors converted them into means and SD using the formula provided by Hozo et al. (23). The standardized mean difference (SMD) was used to evaluate the difference in continuous NLR between irAE and non-irAE groups. If categorized NLR was provided based on the NLR cutoff provided by articles, the authors calculated the ORs and 95% CIs. The authors summarized crude ORs or adjusted ORs for reporting pooled ORs and 95% CIs. Subgroup analysis was performed based on different NLR cutoffs provided by each study and other variables such as ethnicity, ICI type, cancer type, and irAE type were also analyzed. The χ^2^ test combined with the I^2^ statistics were used for evaluating statistical heterogeneity (a *P* value of lower than 0.05 with I^2^≥50% indicated the presence of heterogeneity). If heterogeneity was absent, a fixed-effects model was applied; otherwise, a random-effects model was used. Sensitivity analysis was performed by omitting individual studies one by one to check the influence of each study. Publication bias was evaluated using funnel plots and the Egger test. The statistical analysis was performed by Stata software (version 15.0, Stata Corporation, College Station, TX, USA). P value < 0.05 was set as significant difference.

## Results

### Study selection

**Figure 1:**
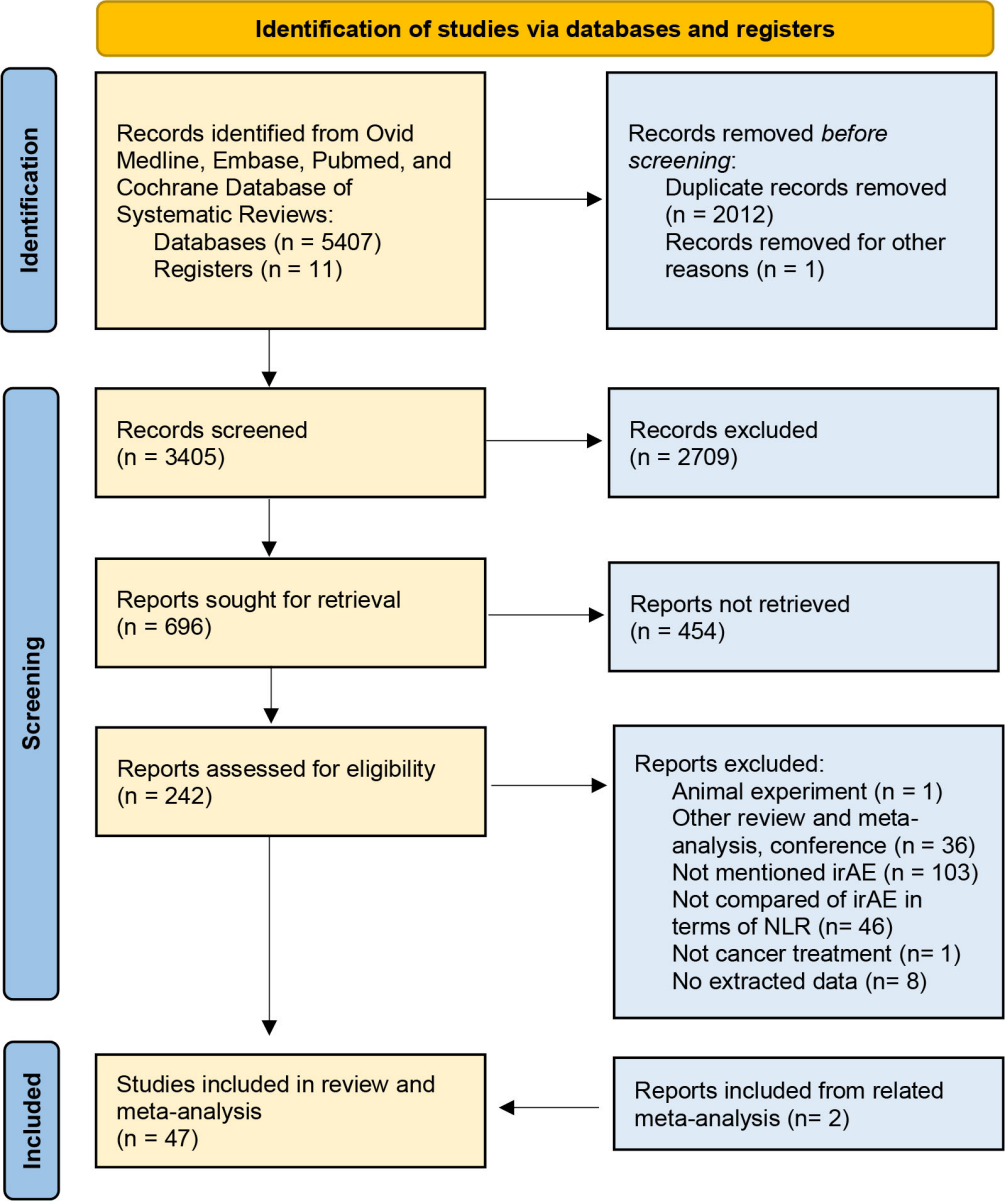
The flowchart of study screening.

A total of 5,418 studies were identified from the four databases using the search strategy. After deleting the duplicated studies, 3,405 studies were screened by titles and abstracts, and 242 studies were eligibility for full-text review. Grey literature was also searched but no additional studies or information were included. Other related reviews and meta-analyses were screened for further inclusion of the studies. Finally, 47 studies (3, 10, 13, 14, 16, 20, 24–64) were included in our review (**Figure 1**).

### Characteristics of included studies

**Table 1** shows the characteristics of the 47 included studies. All studies were published between 2018 to 2023, with recruitment periods between 2014 to 2021. Thirty-three studies were reported from Asia, with 16 from Japan, 15 from China, and the remaining two from Korea and Singapore, respectively. The other 14 studies were from the United States (n=7), Australia (n=2), Italy, Germany, Canada, Belgium, and Spain (each reporting one study). Regarding cancer type, 21 studies only included lung cancer patients, four studies only included hepatocellular carcinoma, three studies only included renal cell or urothelial carcinoma, two studies only included melanoma, one study only included head and neck squamous cell carcinoma, one study only included gastric cancer, and the remaining 15 studies contained mixed cancers. In terms of the types of ICIs, 25 studies only evaluated the irAEs of PD-1 inhibitors, four studies only evaluated the irAEs of Atezolizumab (a PD-L1 inhibitor), and the remaining 18 studies contained PD-1/PD-L1 and CTLA-4 inhibitors. Of the 47 studies, 35 evaluated all types of irAEs, three studies only reported ir-SAE, three studies only reported immune-related pneumonitis and interstitial lung disease, three studies reported cardiovascular adverse events, and the remaining three studies reported colitis, thrombosis, and hypothyroidism, respectively. Regarding peripheral NLR, 44 studies evaluated the predictive value of baseline NLR, three studies assessed the value of NLR 2-6 weeks after ICI treatment, and four studies evaluated the predictive value of dynamic NLR in irAEs. Other peripheral blood biomarkers were also evaluated, of which 21 studies discussed the predictive value of platelet to lymphocyte ratio.

**Table 1:** Characteristics of the included studies.

Author	Published year	Country	Recruitment period	Cancer	Immune checkpoint inhibitors	irAE type	NLR collected time	NLR data type	NLR cutoff	Other peripheral blood biomarker
**Owen, Dwight H. et al.**	2018	United States	2014-2016	lung cancer	Nivolumab, Pembrolizumab, or Atezolizumab	All types of irAE, pneumonitis specified	baseline	categorized	5	ALI, PLR
**Eun, Y. et al**	2019	Korea	2015-2017	lung cancer, melanoma, lymphoma and others	Pembrolizumab	All types of irAE	baseline	both	3	WBC, ANC
**Fukihara, Jun et al**	2019	Japan	2016-2018	lung cancer	Nivolumab or Pembrolizumab	pneumonitis	baseline	continuous	NG	WBC, CRP
**Nakamura, Y. et al**	2019	Japan	2014-2017	melanoma	Nivolumab or Pembrolizumab	All types of irAE	baseline	continuous	NG	WBC, ANC, ALC, AMC, AEC
**Nakanishi, Yu et al**	2019	Japan	2015-2017	lung cancer	Nivolumab or Pembrolizumab	interstitial lung disease	baseline	continuous	NG	WBC, ANC, ALC, LDH, CRP
**Pavan, A. et al**	2019	Italy	2013-2018	lung cancer	Nivolumab, Pembrolizumab, or Atezolizumab	All types of irAE	baseline	categorized	3	PLR
**Drobni, Z, D. et al**	2020	United States	2013-2019	lung cancer, melanoma, renal cell carcinoma, head and neck carcinoma, and others	Not specified, including anti–PD-1, anti-PD-L1, anti-CTLA4 inhibitors	myocarditis	baseline	continuous	NG	WBC, ANC, ALC, AMC
**Grover, S. et al**	2020	United States	2011-2017	melanoma	Nivolumab, Pembrolizumab, or Ipilimumab	colitis	baseline	categorized	5 and 3	AEC
**Kichenadasse, G. et al**	2020	Australia	NG	lung cancer	Atezolizumab	All types of irAE	baseline	continuous	NG	CRP
**Kobayashi, Kazuo et al**	2020	Japan	2016-2018	renal Cell Carcinoma	Nivolumab	All types of irAE	baseline	categorized	3.4	WBC, ANC, PLT, PLR
**Moey, M. Y. Y. et al**	2020	United States	2015-2018	lung cancer	Nivolumab, Pembrolizumab, or Atezolizumab	major adverse cardiac events	baseline	continuous	NG	WBC, CRP
**Ogihara, K. et al**	2020	Japan	2017-2019	urothelial carcinoma	Pembrolizumab	ir-SAE	baseline	categorized	3.35	NG
**Peng, L. et al**	2020	China	2017-2019	lung cancer	Nivolumab, Pembrolizumab, Toripalimab, or Sintilimab	All types of irAE	baseline	categorized	5	LDH
**Daniello, L. et al**	2021	Germany	2012-2020	lung cancer	Nivolumab, Pembrolizumab, Atezolizumab, or Durvalumab	All types of irAE	baseline	both	5	NG
**Egami, S. et al**	2021	Japan	2015-2018	lung cancer	Nivolumab	All types of irAE	after 2 weeks of therapy	categorized	4.3	WBC, ANC, ALC, AMC, LMR
**Egami, S. et al-2**	2021	Japan	2015-2018	lung cancer	Pembrolizumab	All types of irAE	baseline	categorized	2.3	ALC, LMR, PLR
**Fan, X. et al**	2021	China	2018-2020	gastric and colorectal cancers	Not specified, including anti–PD-1 inhibitor	All types of irAE	baseline	categorized	5	MLR PLR
**Fujimoto, A. et al**	2021	Japan	2016-2020	lung cancer	Nivolumab, Pembrolizumab, or Atezolizumab	All types of irAE	baseline	both	2.86	ANC, ALC
**Ksienski, D. et al**	2021	Canada	2017-2019	lung cancer	Pembrolizumab	All types of irAE	baseline	categorized	6.4	PLR
**Lee, P. Y. et al**	2021	Singapore	2014-2019	lung cancer, renal cell carcinoma, nasopharyngeal carcinoma, melanoma	Nivolumab, Pembrolizumab, Atezolizumab, Avelumab, Durvalumab, or Tremelimumab	All types of irAE	baseline and after 6 weeks of therapy	both	5 and 3	ANC, ALC, PLR
**Lin, X. et al**	2021	China	2016-2021	lung cancer	Not specified, including anti–PD-1, anti-PD-L1 inhibitor	pneumonitis	baseline	categorized	5.38	ANC,AEC,IL-2,IL-4,IFN-γ,TNF-a
**Liu, W. et al**	2021	China	2017-2020	lung cancer	Nivolumab or Pembrolizumab	All types of irAE	baseline	continuous	NG	PLR, ANC
**Matsukane, R. et al**	2021	Japan	2018-2020	lung cancer, renal cell carcinoma, head and neck carcinoma, melanoma	Nivolumab or Pembrolizumab	All types of irAE	baseline	categorized	3.8	NG
**Michailidou, D. et al**	2021	United States	2018	lung, skin, genitourinary, gastrointestinal, sarcoma, hematological malignancy, head and neck, breast carcer	Nivolumab, Pembrolizumab, Cemiplimab, Atezolizumab, Durvalumab, Avelumab, Ipilimumab, or Tremelimumab	All types of irAE	baseline	categorized	5.3	ANC, ALC, AMC, MLR, PLR
**Roussel, E. et al**	2021	Belgium	2012-2020	renal cell carcinoma	Nivolumab	All types of irAE	baseline	both	3	ANC, CRP, LDH
**Ruan, D. Y. et al**	2021	China	2016-2017	advanced gastric cancer	Toripalimab	All types of irAE	baseline and dynamic of NLR	categorized	2.7	PLR, LMR
**Ruste, V. et al**	2021	China	2016-2017	melanoma and lung cancer	Toripalimab	All types of irAE	baseline	continuous	NG	CRP, LDH, ADC, ANC, AEC
**Shi, Y. et al**	2021	China	2015-2020	lung cancer	Not specified, including anti–PD-1, anti-PD-L1, anti-CTLA4 inhibitors	All types of irAE	baseline	categorized	5	ANC, AEC, ALC, LDH, CRP
**Abed, A. et al**	2022	Australia	2018-2020	lung cancer	Nivolumab, Pembrolizumab, or Atezolizumab	All types of irAE	baseline	categorized	5	ALC, PLR
**Cánovas, M. S. et al**	2022	Spain	2015-2019	melanoma and lung cancer	Nivolumab, Pembrolizumab, Atezolizumab, or Durvalumab	thrombosis	baseline	categorized	3.01 and 4.55	NG
**Gannichida, A. et al**	2022	Japan	2015-2019	lung cancer, renal cell carcinoma, head and neck carcinoma, melanoma, gastric cancer	Nivolumab	hypothyroidism	baseline	categorized	3.5 and 5	NG
**Lu, X. et al**	2022	China	2019-2021	lung cancer	Not specified, including anti–PD-1 inhibitor	All types of irAE	baseline	categorized	3.56	PLR
**Ma, Y. et al**	2022	China	2017-2019	lung, esophageal carcinoma, liver cancer, head and neck cancer, genital system cancer, colorectal cancer, gastric carcinoma, urogenital carcinoma, cutaneous soft tissue carcinoma, melanoma, gallbladder carcinoma and bile duct carcinoma	Nivolumab, Atezolizumab, Sintilimab, or Camrelizumab	All types of irAE	baseline	categorized	8.58	PLR, AEC
**Matsuo, M. et al**	2022	Japan	2017-2020	head and neck squamous cell carcinoma	Nivolumab	All types of irAE	baseline	categorized	6.505	CRP, PLR, CAR
**Sonehara, K. et al**	2022	Japan	2016-2021	lung cancer	Nivolumab, Pembrolizumab, or Atezolizumab	All types of irAE	baseline	continuous	NG	ALB, PLR
**Tada, T. et al**	2022	Japan	2020-2021	hepatocellular carcinoma	Atezolizumab	All types of irAE	baseline	categorized	3	NG
**Takada, S. et al**	2022	Japan	2017-2020	gastric and renal cancer	Nivolumab	All types of irAE	baseline and dynamic of NLR	categorized	4.3	PLR
**Wu, S. et al**	2022	China	2018-2022	lung stomach esophageal liver colorectal and other	Nivolumab, Pembrolizumab, Camrelizumab, Atezolizumab, Sintilimab, Toripalimab, Tislelizumab, or Durvalumab	cardiovascular adverse events	baseline	categorized	3	NG
**Wu, Y. L. et al**	2022	United States	2019-2022	hepatocellular carcinoma	Atezolizumab	All types of irAE	baseline	categorized	5	PLR
**Zhang, Z. et al**	2022	China	2016-2022	esophageal, gastric, or colon cancer	Nivolumab, Pembrolizumab, Zimberelimab, Camrelizumab, Sintilimab, Tislelizumab, Toripalimab, Atezolizumab, Sugemalimab, Envafolimab, Nivolumab, Ipilimumab, or Cadolinimab	All types of irAE	baseline and 2-3 weeks after (C2)	continuous	NG	PLR, LMR
**Zhao, L. et al**	2022	China	2018-2020	lung, esophagus, gastrointestinal	Nivolumab, Pembrolizumab, Camrelizumab, or Toripalimab	ir-SAE	baseline	continuous	NG	PLR, LDH
**Zheng, X. et al**	2022	China	2018-2021	hepatocellular carcinoma	Camrelizumab	All types of irAE	baseline	categorized	2.22	NG
**Fujimoto, A. et al**	2023	Japan	2018-2021	lung cancer	Nivolumab, Pembrolizumab, Ipilimumab, or Atezolizumab	All types of irAE	baseline	categorized	3	WBC, PLT, PLR
**Lin, X. et al**	2023	China	2018-2021	lung cancer	Pembrolizumab, Nivolumab, Camrelizumab or Sintilimab	All types of irAE	dynamic of NLR	categorized	0.2	NG
**Ochi, H. et al**	2023	Japan	2020-2021	hepatocellular carcinoma	Atezolizumab	All types of irAE	baseline	categorized	2.56	NG
**Pan, C. et al**	2023	United States	2015-2017	head and neck squamous cell carcinoma and salivary gland cancer	Pembrolizumab	ir-SAE	baseline	continuous	NG	NG
**Zheng, L. et al**	2023	China	2019-2021	lung cancer	Pembrolizumab, Sintilimab, or Tislelizumab	All types of irAE	dynamic of NLR	categorized	NG	MLR, PLR

NG, not given; irAE, immune-related adverse event; SAE, severe adverse event; WBC, white blood cell count; ANC, absolute neutrophil count; PLR, platelet‐to‐lymphocyte ratio; NLR, neutrophil to lymphocyte ratio; ALC, absolute lymphocyte count; AMC, absolute monocyte count; AEC, absolute eosinophil count; CRP, C-reactive protein; LMR, lymphocyte-to-monocyte ratio; MLR, monocyte-to-lymphocyte ratio; LDH, lactate dehydrogenase.

### Meta-analysis

A total of 11, 491 patients were included in our meta-analysis, with a median number of 115 (range 45 to 1548) enrolled in each study (**Supplementary Table 2**). Sixty-seven percent of patients were male, with a median age ranging from 16 to 89 years. A total of 2,836 irAEs were reported, with a median incidence of 24.5% ranging from 3% to 70%. Twenty-two studies reported the incidence of different subtypes of irAEs (**Supplementary Table 3**). The most common irAE was dermatologic disorders, with an incidence ranging from 6.1% to 77.7%. The incidence of pneumonitis, endocrinopathy, gastrointestinal disorders, and liver injury was 1.7%-33.3%, 4.4%-36.4%, 1.3%-29.2%, and 0.4%-27.5%, respectively.

### Predictive value of continuous NLR for irAE

**Figure 2:**
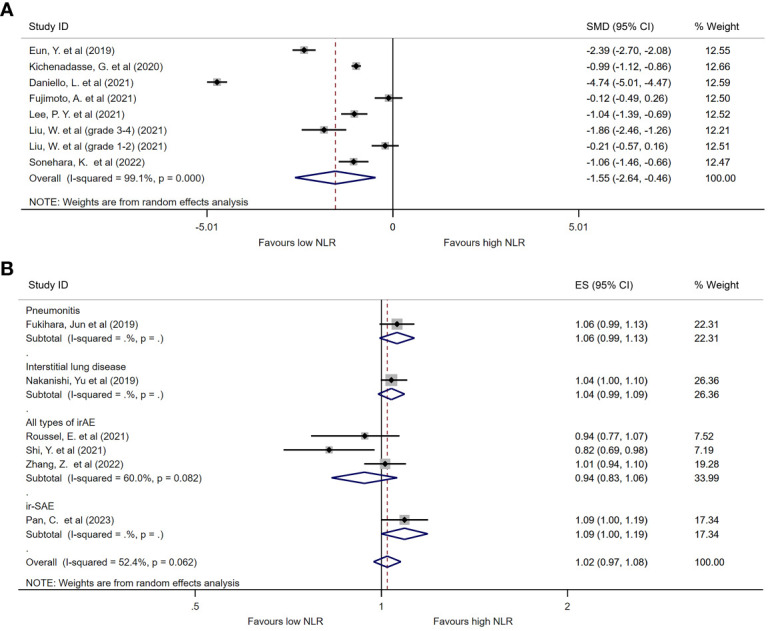
Forest plot comparing continuous NLR between patients who experienced irAEs and those who did not. **(A)** Comparison of mean NLR values between groups. **(B)** Pooled ORs based on continuous NLR data.

Among the identified studies, thirteen listed continuous NLR in both irAE and non-irAE groups. Of these, eight analyzed all types of irAEs (**Table 2**). Median baseline NLR ranged from 2.1 to 10.9 in irAE group, compared with 2.3 to 9 in non-irAE group. Seven studies compared baseline peripheral NLR in all types of irAEs, and were thereby included in the meta-analysis. As shown in **Figure 2A**, the baseline NLR was significantly lower in the irAE group compared with the non-irAE group (SMD=-1.55, 95%CI=-2.64 to -0.46, P=0.006, I^2 =^ 99.1%, random effect model).

**Table 2:** The association between continuous NLR and the incidence of irAE.

Author	Published year	irAE type	NLR collected time	total	irAE group		Non-irAE group	
Comparison of the continuous NLR in two groups					sample	NLR data	sample	NLR data
**Eun, Y. et al**	2019	All types of irAE	baseline	391	67	2.16 (1.10–2.40)	324	3.13 (1.40–3.60)
**Fukihara, Jun et al**	2019	pneumonitis	baseline	170	27	4.2 (1.9-7.2)	143	3.1 (2.1-5.7)
**Nakanishi, Yu et al**	2019	interstitial lung disease	baseline	83	14	4.36 (0.47-99.60)	69	2.72 (0.34-49.74)
**Drobni, Z, D. et al**	2020	myocarditis	baseline	110	55	3.51(2.32-5.40)	55	4.52 (2.47-9.46)
**Kichenadasse, G. et al**	2020	All types of irAE	baseline	1548	340	2.1 (1.6–2.8)	1124	2.3 (1.7–3.3)
**Moey, M. Y. Y. et al**	2020	major adverse cardiac events	baseline	196	23	10.9 (8.3)	173	8.1 (9.0)
**Daniello, L. et al**	2021	All types of irAE	baseline	894	198	7 (0.7)	696	9 (0.3)
**Fujimoto, A. et al**	2021	All types of irAE	baseline	115	45	2.8 (0.9–12.0)	70	4.1 (0.8–10.7)
**Lee, P. Y. et al**	2021	All types of irAE	baseline	147	91	3.12 (2.22–5.93)	56	3.77 (2.92–7.49)
**Lee, P. Y. et al**	2021	All types of irAE	6 weeks after therapy	147	91	3.20 (2.23–5.08)	56	4.21 (2.48–6.83)
**Liu, W. et al**	2021	All types of irAE (grade3-4)	baseline	150	15	3.22 (2.24–4.61)	93	4.25 (3.06–10.49)
**Liu, W. et al**	2021	All types of irAE (grade1-2)	baseline	150	42	4.44 (3.24–8.86)	93	4.25 (3.06–10.49)
**Ruste, V. et al**	2021	All types of irAE	baseline	1187	34	4.78 (1.42-28.5)	807	
**Sonehara, K. et al**	2022	All types of irAE	baseline	113	44	3.84 (1.48–8.67)	69	4.38 (0.55–48.77)
**Zhao, L. et al**	2022	ir-SAE	baseline	168	42	4.0 (2.5–6.4)	236	3.0 (2.3–3.8)
Extracted OR for predicting irAE in terms of continuous NLR					Variable	Extracted OR	Lower 95% CI	Higher 95% CI
**Fukihara, Jun et al**	2019	pneumonitis	baseline	170	continuous NLR	1.06	0.993	1.131
**Nakamura, Y. et al**	2019	All types of irAE	baseline	45	continuous NLR for vitiligo irAE	0.348	0.118	1.025
**Nakanishi, Yu et al**	2019	interstitial lung disease	baseline	83	continuous NLR	1.03783	0.99754	1.1014
**Roussel, E. et al**	2021	All types of irAE	baseline	113	continuous NLR	0.94	0.77	1.07
**Shi, Y. et al**	2021	All types of irAE	baseline	103	continuous NLR	0.823	0.695	0.975
**Zhang, Z. et al**	2022	All types of irAE	2-3 weeks after therapy	234	continuous NLR	0.894	0.801	0.997
**Zhang, Z. et al**	2022	All types of irAE	baseline	234	continuous NLR	1.014	0.938	1.096
**Pan, C. et al**	2023	ir-SAE	baseline	50	continuous for SAE	1.09	1	1.19

irAE, immune-related adverse event; NLR, neutrophil to lymphocyte ratio; OR, odd ratio; CI, confidence interval.

Seven studies evaluated the ORs of continuous NLR in predicting irAEs, of which three reported baseline continuous NLR in all types of irAEs (**Table 2**). Although a trend was found suggesting that the incidence of irAEs could be lower with an increase in continuous NLR, there was no statistical difference observed (OR=0.94, 95%CI=0.83 to 1.06, P=0.311, **Figure 2B**).

### Predictive value of categorized NLR for irAE

Twenty-nine studies categorized NLR to predict irAEs by different cut-offs (**Table 3**). The cut-offs of baseline NLR ranged from 2.3 to 8.58, with seven studies using 3 as the cut-off and twelve studies using 5 as the cut-off. Seventeen studies provided data on the number of lower NLR patients and higher NLR patients in both irAE and non-irAE groups. The pooled ORs for these studies were 0.424 (95%CI=0.308 to 0.584, P<0.001, **Supplementary Figure 1**). Additionally, 17 studies reported calculated ORs of categorized NLR in predicting irAEs, either in univariate or adjusted methods. The pooled ORs for these studies were 0.61 (95%CI=0.39 to 0.94, P=0.027, **Supplementary Figure 2**). Combining all studies reporting categorized NLR to predict irAEs, we found that lower NLR was associated with a higher incidence of irAEs (OR=0.55, 95%CI=0.41-0.73, I^2 =^ 71.1%, P<0.001, **Supplementary Figure 3**).

**Table 3:** The association between categorized NLR and the incidence of irAE.

Author	Published year	irAE type	NLR collected time	total	NLR cutoff	irAE group		Non-irAE group	
Comparison of the categorized NLR in two groups						lower NLR	higher NLR	lower NLR	higher NLR
**Owen, Dwight H. et al.**	2018	All types of irAE	baseline	91	5	12	15	29	35
**Eun, Y. et al**	2019	All types of irAE	baseline	391	3	58	9	216	108
**Pavan, A. et al**	2019	All types of irAE	baseline	184	3	32	26	42	74
**Grover, S. et al**	2020	colitis	baseline	213	5	31	6	121	55
**Grover, S. et al**	2020	colitis	baseline	213	3	19	18	71	107
**Ogihara, K. et al**	2020	ir-SAE	baseline	78	3.35	14	5	31	28
**Peng, L. et al**	2020	All types of irAE	baseline	102	5	32	7	12	51
**Daniello, L. et al**	2021	All types of irAE	baseline	894	5	98	93	233	444
**Fan, X. et al**	2021	All types of irAE	baseline	111	5	25	5	69	12
**Fujimoto, A. et al**	2021	All types of irAE	baseline	115	2.86	25	20	20	50
**Ruan, D. Y. et al**	2021	All types of irAE	baseline	58	2.7	7	7	6	10
**Shi, Y. et al**	2021	All types of irAE	baseline	103	5	29	9	40	25
**Gannichida, A. et al**	2022	hypothyroidism	baseline	104	3.5	16	5	44	39
**Gannichida, A. et al**	2022	hypothyroidism	baseline	104	5	20	1	58	25
**Lu, X. et al**	2022	All types of irAE	baseline	133	3.56	12	10	56	55
**Ma, Y. et al**	2022	All types of irAE	baseline	95	8.58	51	2	35	7
**Matsuo, M. et al**	2022	All types of irAE	baseline	164	6.505	45	7	58	54
**Wu, S. et al**	2022	cardiovascular adverse events	baseline	495	3	42	22	176	255
**Wu, Y. L. et al**	2022	All types of irAE	baseline	296	5	49	12	176	44
Extracted OR for predicting irAE in terms of categorized NLR						Variable	Extracted OR	Lower 95% CI	Higher 95% CI
**Pavan, A. et al**	2019	All types of irAE	baseline	184	3	low vs high NLR	1.7	0.8	3.3
**Grover, S. et al**	2020	colitis	baseline	213	5	high vs low NLR	0.34	0.1	0.9
**Kobayashi, Kazuo et al**	2020	All types of irAE	baseline	53	3.4	low vs high NLR	3.21	0.55	18.76
**Peng, L. et al**	2020	All types of irAE	baseline	102	5	high vs low NLR	0.04	0.01	0.13
**Egami, S. et al**	2021	All types of irAE	2 weeks after therapy	171	4.3	high vs low NLR	0.57	0.3	1.08
**Egami, S. et al-2**	2021	All types of irAE	baseline	92	2.3	high vs low NLR	5.99	1.73	20.74
**Fujimoto, A. et al**	2021	All types of irAE	baseline	115	2.86	low vs high NLR	2.69	1.21	6.01
**Ksienski, D. et al**	2021	All types of irAE	baseline	220	6.4	high vs low NLR	0.79	0.32	1.94
**Lee, P. Y. et al**	2021	All types of irAE	baseline	147	3	low vs high NLR	2.5	1.2	5.22
**Lee, P. Y. et al**	2021	All types of irAE	baseline	147	5	low vs high NLR	1.5	0.74	3.05
**Lin, X. et al**	2021	grade 3-4 pneumonitis	baseline	174	5.38	high vs low NLR	1.28	0.25	6.7
**Matsukane, R. et al**	2021	All types of irAE	baseline	275	3.8	high vs low NLR	1.18	0.67	2.06
**Michailidou, D. et al**	2021	All types of irAE	baseline	470	5.3	low vs high NLR	2.07	1.2	3.58
**Abed, A. et al**	2022	All types of irAE	baseline	179	5	low vs high NLR	1.107	0.511	2.401
**Cánovas, M. S. et al**	2022	thrombosis	baseline	665	4	high vs low NLR	2.14	1.24	3.67
**Cánovas, M. S. et al**	2022	thrombosis	baseline	665	3.01	high vs low NLR	3.65	1.25	10.62
**Lu, X. et al**	2022	All types of irAE	baseline	133	3.56	high vs low NLR	1.228	0.452	3.336
**Takada, S. et al**	2022	All types of irAE	baseline	73	4.3	low vs high NLR	0.024	0.0012	0.46
**Fujimoto, A. et al**	2023	All types of irAE	baseline	315	3	low vs high NLR	2.91	1.35	6.27
**Lin, X. et al**	2023	All types of irAE	dynamic of NLR	138	0.2	change-dNLR>0.2	4.355	1.072	19.484

irAE, immune-related adverse event; NLR, neutrophil to lymphocyte ratio; OR, odd ratio; CI, confidence interval.

### Subgroup analysis

**Figure 3:**
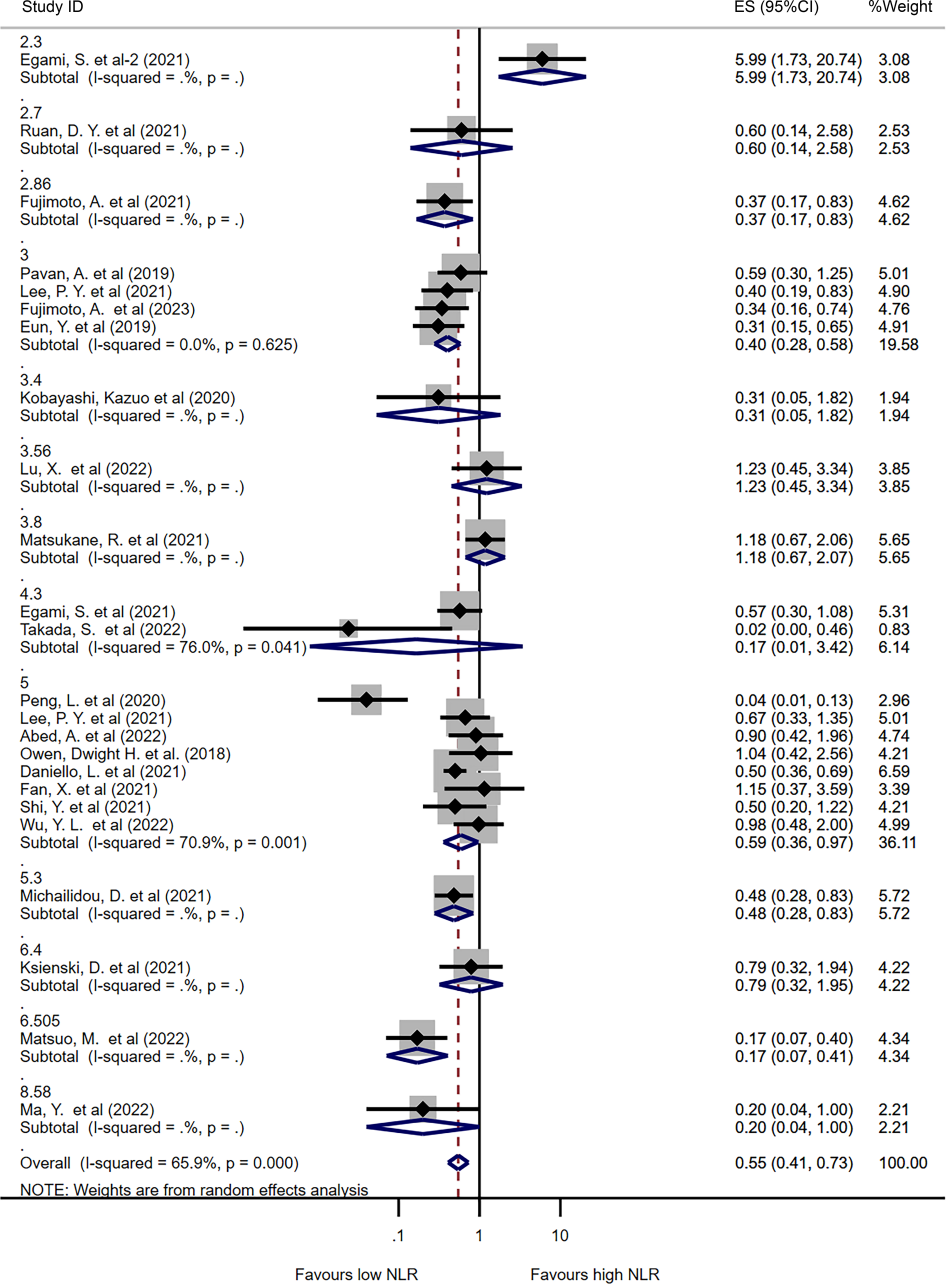
Forest plot comparing categorized NLR for overall irAEs using different cut-off values. Pooled ORs are shown for each cut-off.

Subgroup analysis was performed to explore the potential sources of heterogeneity among studies. To avoid bias caused by studies reporting only one type of irAE (36, 42, 43, 47, 61), we included 24 studies that analyzed all types of irAE in the subgroup analysis based on different NLR cut-off values. **Figure 3** presents the pooled ORs for all or individual NLR cut-offs. The overall pooled OR was consistent with the previous result (OR=0.55, 95%CI=0.41-0.73, I^2 =^ 65.9%, P<0.001). Among the different NLR cut-offs, an NLR of 3 or less was associated with a significantly lower incidence of irAEs (OR=0.40, 95%CI=0.28-0.58, I^2 =^ 0%, P<0.001), while an NLR of 5 or less also correlated with a lower incidence of irAEs (OR=0.59, 95%CI=0.36-0.97, I^2 =^ 70.9%, P=0.036).

In addition, subgroup analysis was performed based on cancer type, with most studies including only lung cancer patients (n=12). The results showed that lower NLR values were associated with a higher incidence of irAEs in lung cancer patients (OR=0.60, 95%CI=0.39-0.92, I^2 =^ 72.2%, P=0.018, **Supplementary Figure 4**). Subgroup analysis was also performed based on ICI type, with 12 studies including only PD-1 inhibitors, 6 studies including PD-1 and PD-L1 inhibitors, and 5 studies including PD-1, PD-L1, and CTLA-4 inhibitors. In patients treated with PD-1 inhibitors, lower NLR values were associated with a lower incidence of irAEs (OR=0.52, 95%CI=0.27-0.99, I2 = 80.2%, P=0.046, **Supplementary Figure 5**). Finally, subgroup analysis was performed based on publication area, with studies divided into Asian and non-Asian countries. Similar results were observed in both Asian and non-Asian publications (OR=0.50 for Asian, I^2 =^ 74.3%, P=0.002; OR=0.56 for non-Asian, I^2 =^ 0%, P<0.001, **Supplementary Figure 6**).

### Predictive value of categorized NLR for specified irAE

**Figure 4:**
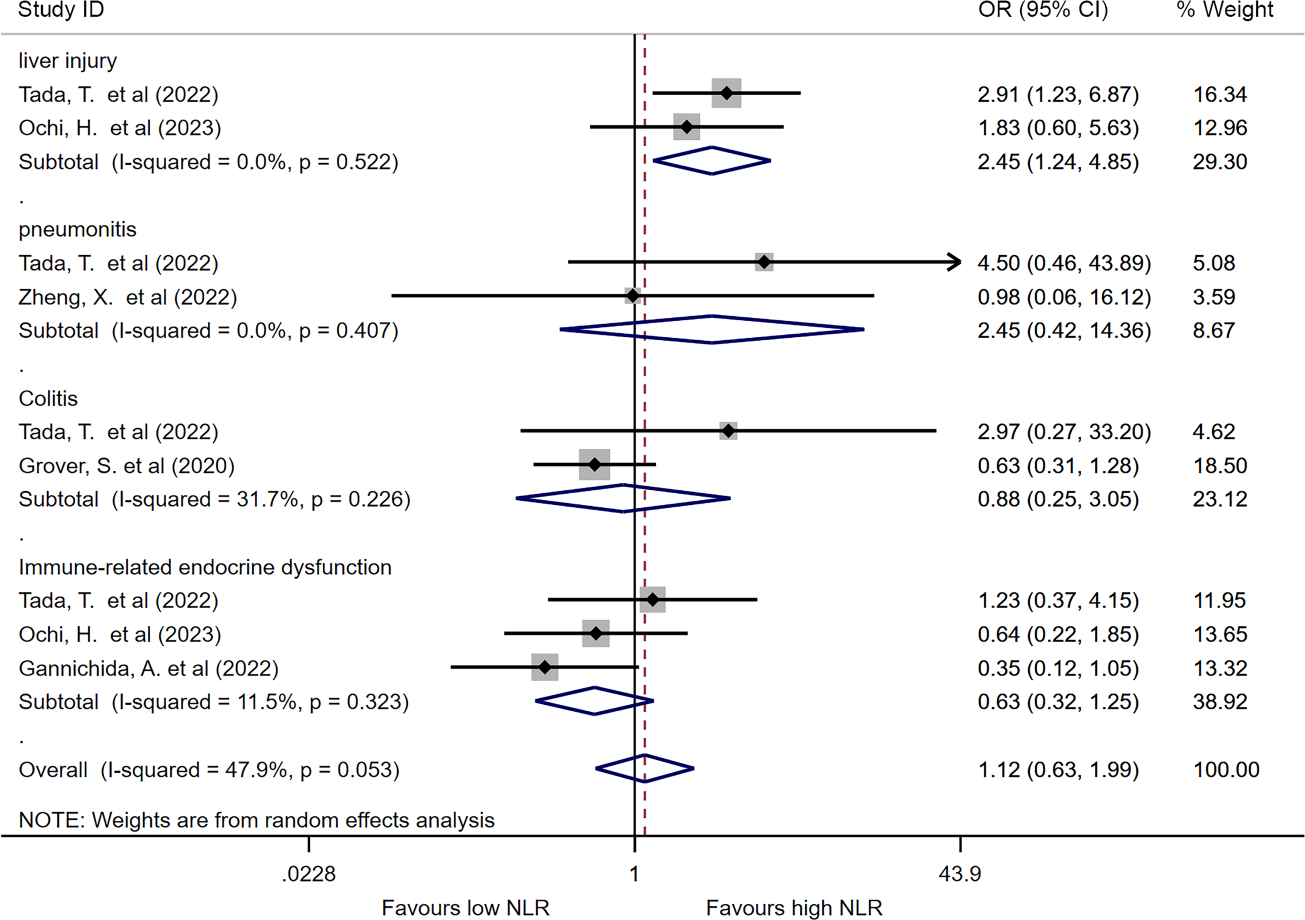
Forest plot comparing categorized NLR for specific irAEs. Pooled ORs are shown for each irAE.

We analyzed the pooled ORs of categorized NLR for specified irAE if two or more studies reported the predictive value. No significant difference was found in terms of pneumonitis, colitis or immune related endocrine dysfunction between irAE and non-irAE group. But interestingly, increased baseline NLR might associate with the increasing incidence of liver injury (OR=2.44, 95%CI=1.23-4.84, I^2 =^ 0%, P=0.010, **Figure 4**).

### Sensitivity analysis

We performed a sensitivity analysis to determine the potential source of heterogeneity. In continuous NLR for predicting irAE, the heterogeneity was influenced remarkably by each study due to the small number of involved studies (**Supplementary Figure 7**). However, in categorized NLR for predicting irAE, only a minority of studies were identified as contributing to the heterogeneity of pooled OR outcomes (**Supplementary Figure 8**). The overall estimate of the pooled ORs would not be significantly influenced when removing any study in turn.

### Quality assessment and publication bias assessment

We considered 40 studies as high quality, while the other 7 studies had NOS scores of 5-6. The funnel plot of the included studies was symmetrical (**Supplementary Figure 9**). For studies comparing continuous NLR between irAE and non-irAE, the Egger’s test suggested no potential publication bias (P=0.744). Similarly, for studies calculating the pooled ORs in continuous or categorized NLR, no potential publication bias was identified by the Egger’s test (P=0.125 and P=0.377, respectively).

## Discussion

In this meta-analysis, our objective was to examine the predictive ability of NLR for irAEs in cancer patients undergoing ICIs treatment. By utilizing a thorough and systematic meta-analysis approach, we evaluated 47 studies comprising 11,491 cancer patients and observed that NLR can serve as a predictor for adverse reactions.

The cancer immunoediting theory describes three stages of interaction between cancer and the immune system during tumorigenesis: elimination, equilibrium, and escape (65). During the elimination stage, both innate and adaptive immunity work together to induce chemokines or recognize tumor antigens to eliminate tumor cells. However, as the tumor progresses, tumor cells survive and reach a balance with the immune system. Eventually, tumor cells evade immune surveillance and gain the upper hand in the tumor microenvironment (TME) (65). The CTLA-4 and PD-1/PD-L1 pathways play a significant role in the escape process of the TME. Some tumors overexpress PD-L1, which increases suppressive co-stimulatory signal production, inhibiting T cell activation and proliferation. In addition, some tumors prompt regulatory T cells (Tregs) to express CTLA-4, leading to downregulation of CD80/CD86 expression in antigen-presenting cells, resulting in reduced production of cytokines such as interleukin 2, which affects the body’s anti-tumor capacity (66). While the mechanism of irAEs is still unclear, some studies have found that T cells are heavily infiltrated in tumor tissues of patients with irAEs (67). According to current findings, the mechanism of irAEs may include the over-activation of effector T cells caused by the inhibition of CTLA-4, PD-1 or PD-L1, reduced function of regulatory T cells, massive release of tumor necrosis factor and gamma interferon, toxic effects of neutrophils and macrophages, and production of antibodies by B cells (68).

During tumorigenesis, neutrophils can produce cytokines and growth factors that lead to immune escape of tumors, and therefore promote tumor growth, invasion, and metastasis (69). On the other hand, lymphocytes, such as T cells, play a crucial role in anti-tumor immune response, suppressing tumor growth (70). Besides, elevated neutrophils can inhibit the anti-tumor function of lymphocytes, leading to weakened attack on mutated cells (71). The NLR imbalance can directly decrease the anti-tumor immune response, accelerating tumor invasion and metastasis, resulting in poor prognosis (32). However, the role of NLR in predicting irAEs is not fully understood. Previous studies have suggested that immune-related toxicities are a group of heterogeneous manifestations, with distinct immunopathogenic mechanisms and different histopathological phenotypes in each involved organ (63, 64). In our study, we found that a lower NLR indicated a higher incidence of all kinds of irAEs. Specifically, we observed that a higher NLR was associated with an increased incidence of immune-related liver injury, although only two studies were included for analysis (28, 32). These studies focused on ICI treatment in hepatocellular carcinoma patients, who may have underlying liver disease leading to distinct results. Wu et al. (62) suggested that a higher NLR could be associated with severe disease burden and liver dysfunction, with patients having an NLR >5 exhibiting higher incidence of elevated alpha-fetoprotein and neoplastic portal vein hypertension. However, more studies should be conducted to investigate the relationship between NLR and distinct types of irAEs.

The optimal cut-off value for NLR varied among the studies included in our analysis. As our studies presented, most studies used a cut-off of 5 to categorize high and low NLR, followed by 3. The criteria used to identify the best cut-off for NLR differed across studies, with some using median values or diagnostic experiments. Additionally, the cut-off of NLR was determined by various factors, including the study participants, tumor type, ICI agents, and risk factors considered, leading to heterogeneity in the analysis of the impact of NLR. Nonetheless, most studies suggested that lower NLR was associated with a higher incidence of irAEs, with this trend or significant difference being observed in the majority of studies included in the meta-analysis.

While our meta-analysis only considered baseline NLR as an indicator for predicting irAEs, some other studies have also investigated the predictive value of dynamic or post-treatment NLR (13, 33, 38, 46, 57, 60, 63). However, these studies have not found dynamic or post-treatment NLR to be a better predictor than baseline NLR, although more recent studies have focused on studying dynamic NLR as an independent predictor. Despite the heterogeneity resulting from potential risk factors, our conclusion was still consistent with the observed trend across studies.

Our study has several strengths. Firstly, to the best of our knowledge, this is the most comprehensive meta-analysis that includes 47 studies investigating the predictive value of peripheral NLR for irAEs. Secondly, our analysis not only compared NLR in categorized conditions based on cut-off values but also examined the impact of baseline continuous NLR in predicting irAEs, thereby strengthening our conclusions. Thirdly, we performed sufficient subgroup analyses based on different NLR cut-off values, cancer types, ICI agents, and ethnicities, and demonstrated that there were differences in the predictive value of NLR between overall irAEs and specific irAEs, such as immune-related liver injury, which has not been reported in previous studies. Additionally, all the included studies were of moderate to high quality, and sensitivity analyses showed robust results.

There are some limitations to our study that need to be considered. Firstly, as our meta-analysis is based on retrospective studies, there is a possibility of heterogeneity and publication bias among the studies. Future studies based on prospective design or individual patient data may provide more robust results. Secondly, due to the variability of cut-offs of NLR used in different studies, we could not determine a consensus on the best cut-off value based on our analysis, which may limit clinical guidance. Thirdly, although our study included a relatively comprehensive set of studies, negative results from non-publication studies could lead to selection bias. Finally, despite the differences in predictive value for different subtypes of irAEs, more studies are needed to investigate specific irAEs as there are currently limited reports available.

## Conclusion

In summary, our meta-analysis revealed a significant association between lower baseline NLR and increased risk of irAEs. However, the predictive value of NLR varied among different types of irAEs, indicating a need for further subgroup analysis in evaluating the efficacy of peripheral biomarkers. The most frequently used cut-offs for NLR were 3 and 5, but a consensus on the best “cut-off” is required for future clinical guidance. Overall, our findings suggest that NLR can serve as a valuable tool in predicting irAEs, and further studies are necessary to explore its potential role in personalized immunotherapy management.

## Data availability statement

The original contributions presented in the study are included in the article/**Supplementary Material**. Further inquiries can be directed to the corresponding authors.

## Author contributions

Design of the meta-analysis: WZ, YT, YL and JL. Literature screening: WZ, and YT. Quality assessment: WZ and YL. Statistics analysis: WZ. Write and revise: WZ, YT, YL, and JL. All authors contributed to the article and approved the submitted version.

## Supplementary material

Supplementary Figure 1 Forest plot comparing pooled ORs based on raw data reported in the original studies.

Supplementary Figure 2 Forest plot comparing pooled ORs based on adjusted ORs reported in the original studies.

Supplementary Figure 3 Forest plot comparing pooled ORs based on both adjusted and crude ORs reported in the original studies.

Supplementary Figure 4 Subgroup analysis of the pooled ORs in terms of cancer type.

Supplementary Figure 5 Subgroup analysis of the pooled ORs in terms of ICI type.

Supplementary Figure 6 Subgroup analysis of the pooled ORs in terms of publication area.

Supplementary Figure 7 Sensitivity analysis of included studies for continuous NLR data.

Supplementary Figure 8 Sensitivity analysis of included studies for categorized NLR data.

Supplementary Figure 9 Funnel plot showing the publication bias assessment of the included studies.
