# Supplementary material for: Neutrophil to Lymphocyte ratio as a predictor for immune-related adverse events in cancer patients treated with immune checkpoint inhibitors: a systematic review and meta-analysis
Source: Front Immunol. 2023 Aug 9;14:1234142. doi: 10.3389/fimmu.2023.1234142 (PMC10445236; doi:10.3389/fimmu.2023.1234142)
Supplement: Supplementary file 1 [file Table_1.docx]

Supplementary Table 1 Search strategy of including studies

| Search strategy (example as Ovid Medline) |
| --- |
| 1 Immune Checkpoint Inhibitors/  2 immune checkpoint inhibitor.mp.  3 immune checkpoint inhibitors.mp.  4 immune checkpoint blockade.mp.  5 immune checkpoint therapy.mp.  6 immunotherapy.mp.  7 immune related adverse events.mp.  8 immune related adverse event.mp.  9 or/1-8  10 CTLA-4.mp.  11 CTLA4.mp.  12 cytotoxic T-lymphocyte-associated protein 4.mp.  13 CD152.mp.  14 PD-1.mp.  15 PD1.mp.  16 programmed cell death protein 1.mp.  17 CD279.mp.  18 PD-L1.mp.  19 PDL1.mp.  20 programmed death-ligand 1.mp.  21 CD274.mp.  22 atezolizumab.mp.  23 avelumab.mp.  24 cemiplimab.mp.  25 durvalumab.mp.  26 ipilimumab.mp.  27 nivolumab.mp.  28 pembrolizumab.mp.  29 or/10-28  30 9 or 29  31 Neutrophils/  32 neutrophil.mp.  33 neutrophils.mp.  34 or/31-33  35 Lymphocytes/  36 lymphocyte.mp.  37 lymphocytes.mp.  38 or/35-37  39 34 and 38  40 NLR.mp.  41 neutrophil to lymphocyte ratio.mp.  42 neutrophil-lymphocyte ratio.mp.  43 or/39-42  44 30 and 43 |
