# Supplementary material for: Neutrophil to Lymphocyte ratio as a predictor for immune-related adverse events in cancer patients treated with immune checkpoint inhibitors: a systematic review and meta-analysis
Source: Front Immunol. 2023 Aug 9;14:1234142. doi: 10.3389/fimmu.2023.1234142 (PMC10445236; doi:10.3389/fimmu.2023.1234142)
Supplement: Supplementary file 2 [file Table_2.docx]

Supplementary Table 2 General data of the included studies

| Author | Published year | irAE type | Total | | | AE group | | | Without AE group | | |
| --- | --- | --- | --- | --- | --- | --- | --- | --- | --- | --- | --- |
|  |  |  | total | male | age | sample | male | age | sample | male | age |
| Owen, Dwight H. et al. | 2018 | All types of irAE | 91 | 39 | 67 (40-87) | 27 | 14 | NG | 64 | 25 | NG |
| Eun, Y. et al | 2019 | All types of irAE | 391 | NG | NG | 67 | 45 | 59 (52–66) | 324 | 202 | 60 (52–69) |
| Fukihara, Jun et al | 2019 | pneumonitis | 170 | NG | NG | 27 | 20 | 67 (58-73) | 143 | 105 | 70 (63-73) |
| Nakamura, Y. et al | 2019 | All types of irAE | 45 | 25 | 69.3 (42-85) | 26 | NG | NG | 19 | NG | NG |
| Nakanishi, Yu et al | 2019 | interstitial lung disease | 83 | 58 | 68 (34-85) | 14 | 10 | 65 (49-80) | 69 | 48 | 68 (34-85) |
| Pavan, A. et al | 2019 | All types of irAE | 184 | 125 | 67 (37–83) | 60 | NG | NG | 124 | NG | NG |
| Drobni, Z, D. et al | 2020 | myocarditis | 110 | NG | NG | 55 | 41 | 67±15 | 55 | 30 | 66±16 |
| Grover, S. et al | 2020 | colitis | 213 | NG | NG | 37 | 22 | NG | 176 | 102 | NG |
| Kichenadasse, G. et al | 2020 | All types of irAE | 1548 | 936 | 64 (57–70) | 340 | 211 | 66 (58–72) | 1124 | 673 | 63 (56–70) |
| Kobayashi, Kazuo et al | 2020 | All types of irAE | 53 | 40 | 67 (41-85) | 24 | NG | NG | 29 | NG | NG |
| Moey, M. Y. Y. et al | 2020 | major adverse cardiac events | 196 | NG | NG | 23 | 15 | 68.7±1.8 | 173 | 99 | 64.3±0.8 |
| Ogihara, K. et al | 2020 | ir-SAE | 78 | NG | NG | 19 | NG | NG | 59 | NG | NG |
| Peng, L. et al | 2020 | All types of irAE | 102 | 87 | NG | 39 | NG | NG | 63 | NG | NG |
| Daniello, L. et al | 2021 | All types of irAE | 894 | NG | NG | 198 | 117 | 65±12 | 696 | 419 | 65±12 |
| Egami, S. et al | 2021 | All types of irAE | 171 | 113 | 64 (56–69) | 73 | NG | NG | 98 | NG | NG |
| Egami, S. et al-2 | 2021 | All types of irAE | 92 | 64 | 60 (34–85) | 45 | NG | NG | 47 | NG | NG |
| Fan, X. et al | 2021 | All types of irAE | 111 | 56 | NG | 30 | NG | NG | 81 | NG | NG |
| Fujimoto, A. et al | 2021 | All types of irAE | 115 | NG | NG | 45 | 32 | 68 (45–87) | 70 | 52 | 69 (44–85) |
| Ksienski, D. et al | 2021 | All types of irAE | 220 | 99 | 70 (63–76) | 89 | NG | NG | 131 | NG | NG |
| Lee, P. Y. et al | 2021 | All types of irAE | 147 | NG | NG | 91 | 60 | 61 (52–70) | 56 | 39 | 63 (56–68) |
| Lin, X. et al | 2021 | pneumonitis | 174 | NG | NG | 87 | 73 | 65 (18–85) | 87 | 66 | 62 (31–83) |
| Liu, W. et al | 2021 | All types of irAE | 150 | 118 | 57.5±10.0 | 15^*^ | 13 | 58.9±9.8 | 93 | 71 | NG |
|  |  |  |  |  |  | 42** | 34 | 57.3±10.0 | NG | NG | NG |
| Matsukane, R. et al | 2021 | All types of irAE | 275 | 203 | 68 (16–89) | 121 | NG | NG | 154 | NG | NG |
| Michailidou, D. et al | 2021 | All types of irAE | 470 | 275 | 65 (56–71) | 156 | NG | NG | 314 | NG | NG |
| Roussel, E. et al | 2021 | All types of irAE | 113 | 46 | 66 (59-73) | 22 | NG | NG | 91 | NG | NG |
| Ruan, D. Y. et al | 2021 | All types of irAE | 58 | 41 | 60 (52–66) | 14 | NG | NG | 44 | NG | NG |
| Ruste, V. et al | 2021 | All types of irAE | 1187 | NG | NG | 34 | 21 | 63 (32-95) | 807 | NG | NG |
|  |  |  |  |  |  | 346 | 191 | 66 (17-93) |  |  |  |
| Shi, Y. et al | 2021 | All types of irAE | 103 | 68 | 66 (61-71) | 38 | NG | 67 (61-72) | 65 | NG | 65 (61-71) |
| Abed, A. et al | 2022 | All types of irAE | 179 | 101 | NG | 77 | NG | NG | 102 | NG | NG |
| Cánovas, M. S. et al | 2022 | thrombosis | 665 | 463 | NG | 56 | NG | NG | 609 | NG | NG |
| Gannichida, A. et al | 2022 | hypothyroidism | 104 | 69 | 69 (32-91) | 21 | 14 | 70 (45-91) | 83 | 55 | 68 (32-88) |
| Lu, X. et al | 2022 | All types of irAE | 133 | 56 | NG | 22 | NG | NG | 111 | NG | NG |
| Ma, Y. et al | 2022 | All types of irAE | 95 | 66 | 62 (30-80) | 53 | NG | NG | 42 | NG | NG |
| Matsuo, M. et al | 2022 | All types of irAE | 164 | 127 | 65 (23-87) | 52 | NG | NG | 112 | NG | NG |
| Sonehara, K. et al | 2022 | All types of irAE | 113 | 91 | 70 (29-87) | 44 | 38 | 71 (29–87) | 69 | 56 | 68 (42–83) |
| Tada, T. et al | 2022 | All types of irAE | 249 | 211 | 73 (68–79) | 148 | NG | NG | 101 | NG | NG |
| Takada, S. et al | 2022 | All types of irAE | 73 | 54 | 67 (60-73) | 51 | NG | NG | 22 | NG | NG |
| Wu, S. et al | 2022 | cardiovascular adverse events | 495 | NG | NG | 64 | 52 | 61.8±10.0 | 431 | 317 | 62.3±10.2 |
| Wu, Y. L. et al | 2022 | All types of irAE | 296 | 245 | 66 (59–73) | 63 | NG | NG | 220 | NG | NG |
| Zhang, Z. et al | 2022 | All types of irAE | 234 | 172 | NG | 139 | NG | NG | 95 | NG | NG |
| Zhao, L. et al | 2022 | ir-SAE | 168 | NG | 60.8±10.2 | 42 | 33 | 60.3±9.6 | 236 | 99 | 60.9±10.4 |
| Zheng, X. et al | 2022 | All types of irAE | 95 | NG | NG | NG | NG | NG | NG | NG | NG |
| Fujimoto, A. et al | 2023 | All types of irAE | 315 | NG | NG | 50 | 41 | 69 (40–80) | 265 | 199 | 68 (42–86) |
| Lin, X. et al | 2023 | All types of irAE | 138 | 113 | NG | 75 | NG | NG | 63 | NG | NG |
| Ochi, H. et al | 2023 | All types of irAE | 242 | NG | NG | 36 | NG | NG | 206 | NG | NG |
| Pan, C. et al | 2023 | ir-SAE | 50 | NG | NG | NG | NG | NG | NG | NG | NG |
| Zheng, L. et al | 2023 | All types of irAE | 139 | 111 | NG | 27 | NG | NG | 112 | NG | NG |
| *：SAE group；  **：not SAE group  Abbreviation: AE：adverse event, NG: not given, SAE: severe adverse event | | | | | | | | | | | |
