# Supplementary material for: Neutrophil to Lymphocyte ratio as a predictor for immune-related adverse events in cancer patients treated with immune checkpoint inhibitors: a systematic review and meta-analysis
Source: Front Immunol. 2023 Aug 9;14:1234142. doi: 10.3389/fimmu.2023.1234142 (PMC10445236; doi:10.3389/fimmu.2023.1234142)
Supplement: Supplementary file 3 [file Table_3.docx]

Supplementary Table 3 The summary of the different types of irAE in the including studies

| Author | Published year | Immune checkpoint inhibitors | total sample | Dermatologic disorders | Pneumonitis | Musculoskeletal disorders | Endocrinopathy | Gastrointestinal disorders | Liver injury | Rheumatic injury | Cardiovascular injury |
| --- | --- | --- | --- | --- | --- | --- | --- | --- | --- | --- | --- |
| Owen, Dwight H. et al. | 2018 | Nivolumab, Pembrolizumab, or Atezolizumab | 27^*^ | 6 | 9 | NG | 7 | 3 | 1 | NG | NG |
| Eun, Y. et al | 2019 | Pembrolizumab | 88 | 49 | 11 | 12 | 7 | 5 | 3 | NG | NG |
| Nakamura, Y. et al | 2019 | Nivolumab or Pembrolizumab | 56 | 15 | 1 | NG | 14 | 6 | 2 | 1 | NG |
| Pavan, A. et al | 2019 | Nivolumab, Pembrolizumab, or Atezolizumab | 60^*^ | 11 | 13 | 5 | 6 | 12 | 12 | NG | NG |
| Kichenadasse, G. et al | 2020 | Atezolizumab | 1124 | 306 | 45 | NG | 85 | 15 | 4 | 8 | NG |
| Kobayashi, Kazuo et al | 2020 | Nivolumab | 24^*^ | 9 | 3 | NG | NG | 7 | 1 | NG | NG |
| Peng, L. et al | 2020 | Nivolumab, Pembrolizumab, Toripalimab, or Sintilimab | 39^*^ | 13 | NG | NG | 7 | NG | 9 | NG | NG |
| Daniello, L. et al | 2021 | Nivolumab, Pembrolizumab, Atezolizumab, or Durvalumab | 232 | 23 | 40 | 38 | 44 | 37 | 33 | NG | 4 |
| Egami, S. et al | 2021 | Nivolumab | 73^*^ | 44 | 2 | NG | 15 | 20 | 3 | NG | NG |
| Egami, S. et al | 2021 | Pembrolizumab | 45^*^ | 35 | 1 | NG | 2 | 9 | 1 | NG | NG |
| Fujimoto, A. et al | 2021 | Nivolumab, Pembrolizumab, or Atezolizumab | 45^*^ | 14 | 8 | NG | 16 | 5 | 2 | NG | 2 |
| Lee, P. Y. et al | 2021 | Nivolumab, Pembrolizumab, Atezolizumab, or Others | 91^*^ | 8 | 5 | NG | 12 | 9 | 11 | 3 | NG |
| Matsukane, R. et al | 2021 | Nivolumab or Pembrolizumab | 166 | 61 | 26 | NG | 17 | 9 | 5 | NG | 2 |
| Michailidou, D. et al | 2021 | Nivolumab, Pembrolizumab, Cemiplimab, or Others | 212 | 13 | 25 | NG | 72 | 58 | 18 | 31 | 1 |
| Shi, Y. et al | 2021 | Not specified, including anti–PD-1, anti-PD-L1, anti-CTLA4 inhibitors | 57 | 16 | 4 | 1 | 12 | 14 | 8 | NG | 3 |
| Abed, A. et al | 2022 | Nivolumab, Pembrolizumab, or Atezolizumab | 156 | 34 | 15 | 20 | 12 | 7 | 10 | NG | NG |
| Ma, Y. et al | 2022 | Nivolumab, Atezolizumab, Sintilimab, or Camrelizumab | 53^*^ | 11 | 7 | NG | 4 | 4 | 11 | NG | 5 |
| Sonehara, K. et al | 2022 | Nivolumab, Pembrolizumab, or Atezolizumab | 44^*^ | 8 | 8 | 1 | 16 | NG | 5 | NG | NG |
| Takada, S. et al | 2022 | Nivolumab | 51^*^ | 18 | 8 | NG | 14 | 5 | 14 | NG | NG |
| Zhang, Z. et al | 2022 | Nivolumab, Pembrolizumab, Camrelizumab, or Others | 260 | 52 | 9 | NG | 49 | 21 | 59 | 35 | 6 |
| Fujimoto, A. et al | 2023 | Nivolumab, Pembrolizumab, Ipilimumab, or Atezolizumab | 50^*^ | 16 | 11 | 2 | 12 | 5 | 5 | 1 | NG |
| Lin, X. et al | 2023 | Pembrolizumab, Nivolumab, Camrelizumab or Sintilimab | 75^*^ | 23 | 19 | NG | 22 | 13 | 19 | NG | 8 |
| Abbreviation: NG: not given, * patient number instead of irAE number | | | | | | | | | | | |
