# Supplementary figures and images for: Neutrophil to Lymphocyte ratio as a predictor for immune-related adverse events in cancer patients treated with immune checkpoint inhibitors: a systematic review and meta-analysis
Source: Front Immunol. 2023 Aug 9;14:1234142. doi: 10.3389/fimmu.2023.1234142 (PMC10445236; doi:10.3389/fimmu.2023.1234142)

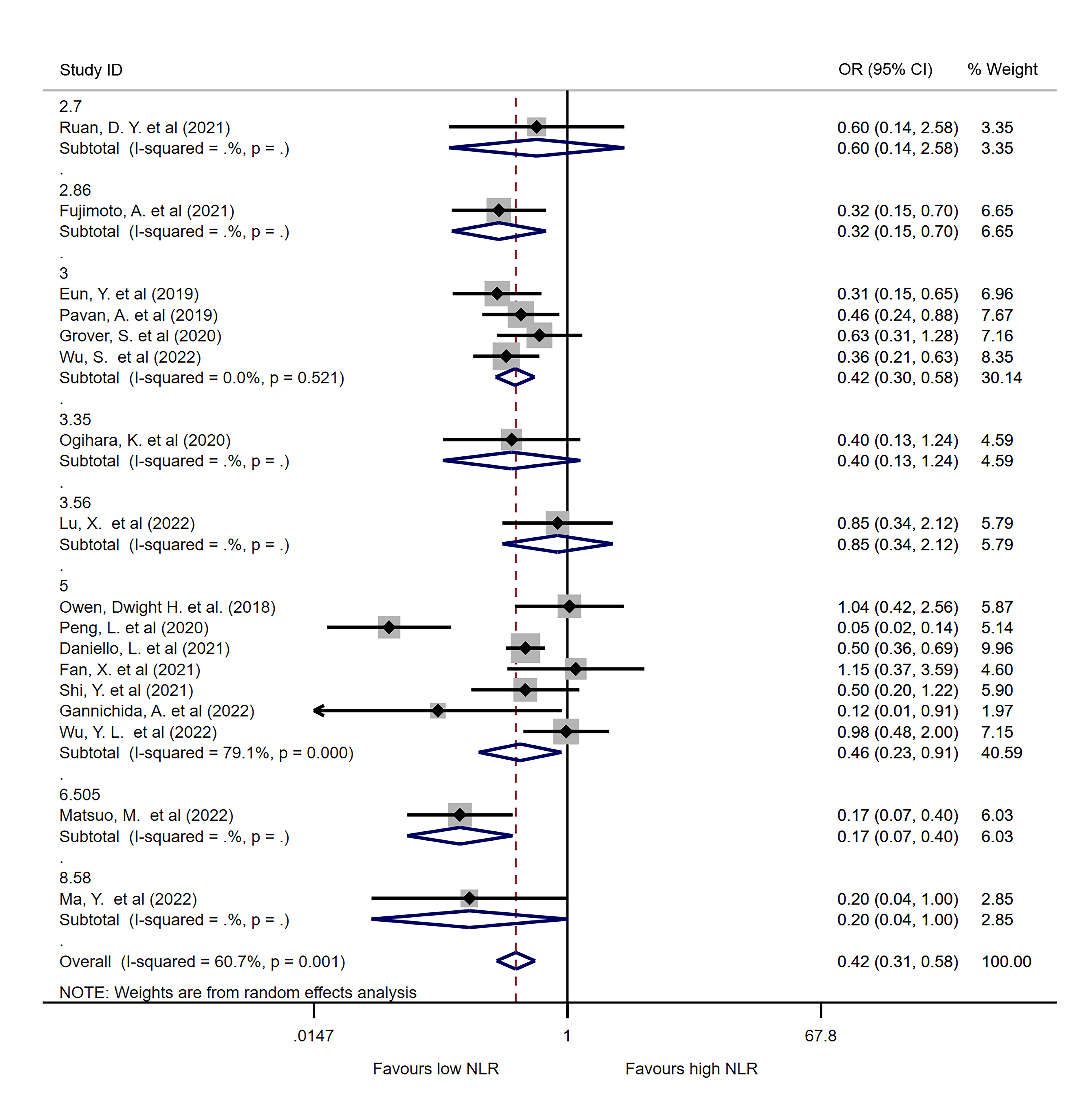

Supplement: Supplementary Figure 1 — Forest plot comparing pooled ORs based on raw data reported in the original studies. [file Image_1.tif]

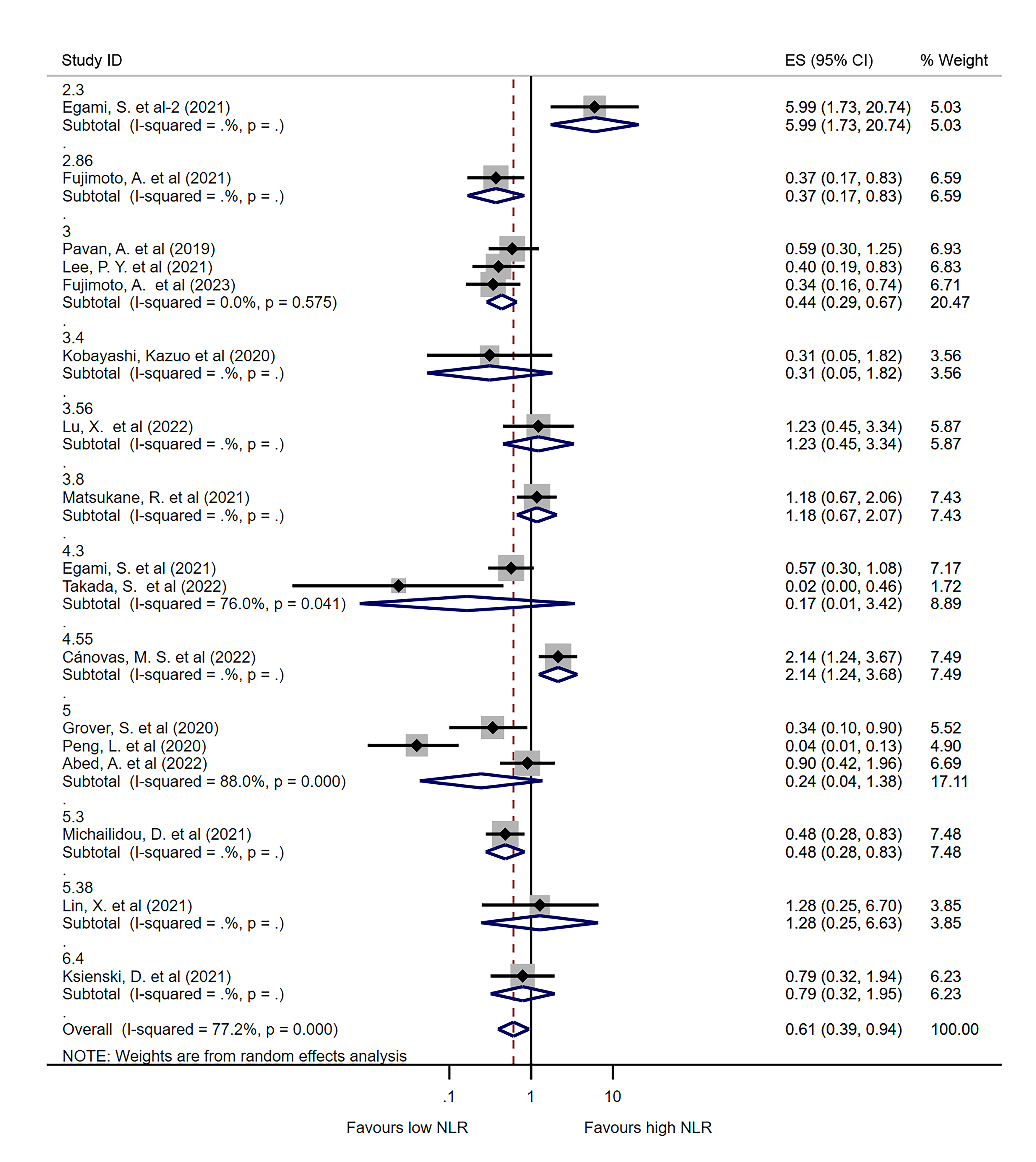

Supplement: Supplementary Figure 2 — Forest plot comparing pooled ORs based on adjusted ORs reported in the original studies. [file Image_2.tif]

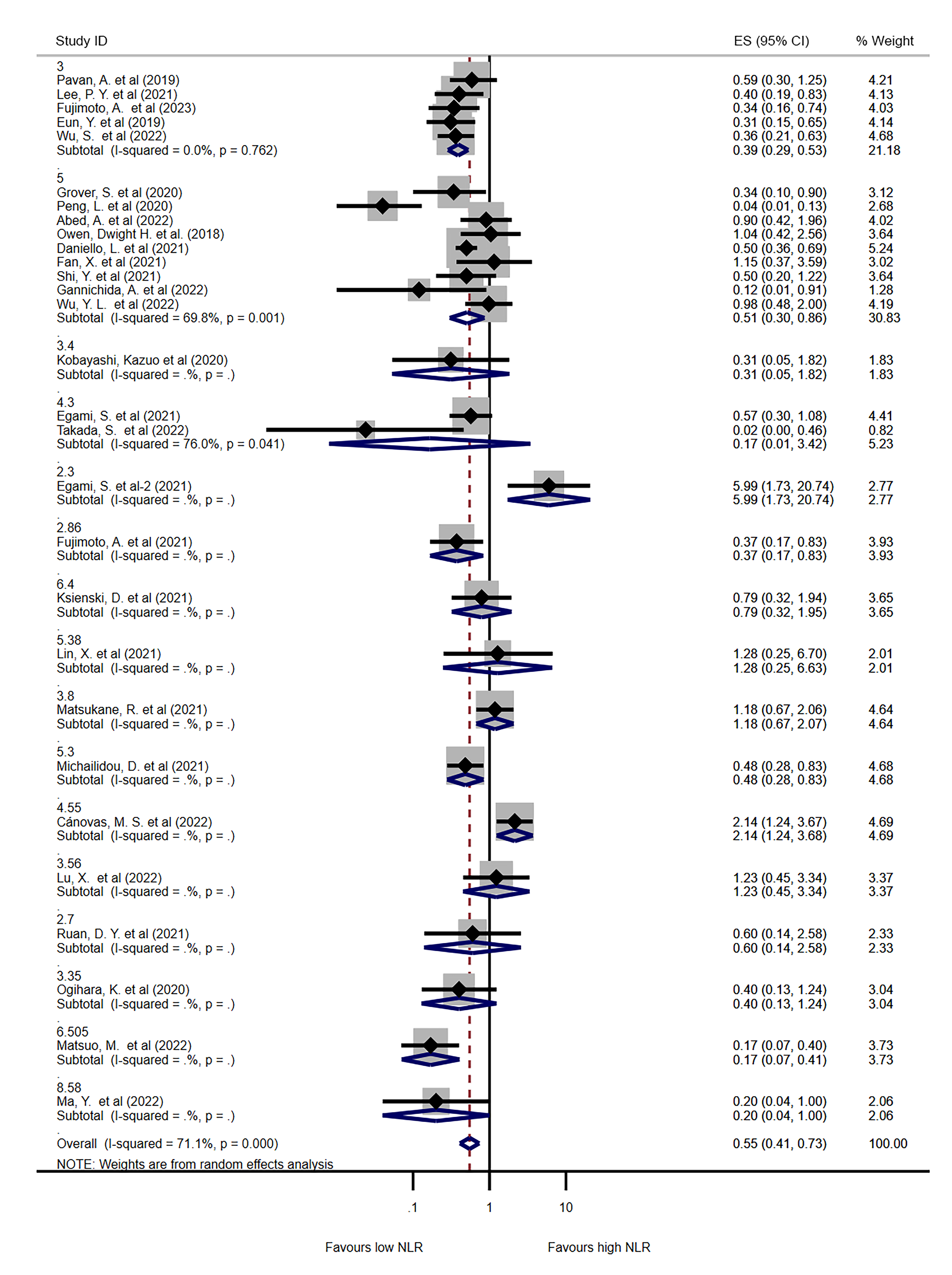

Supplement: Supplementary Figure 3 — Forest plot comparing pooled ORs based on both adjusted and crude ORs reported in the original studies. [file Image_3.tif]

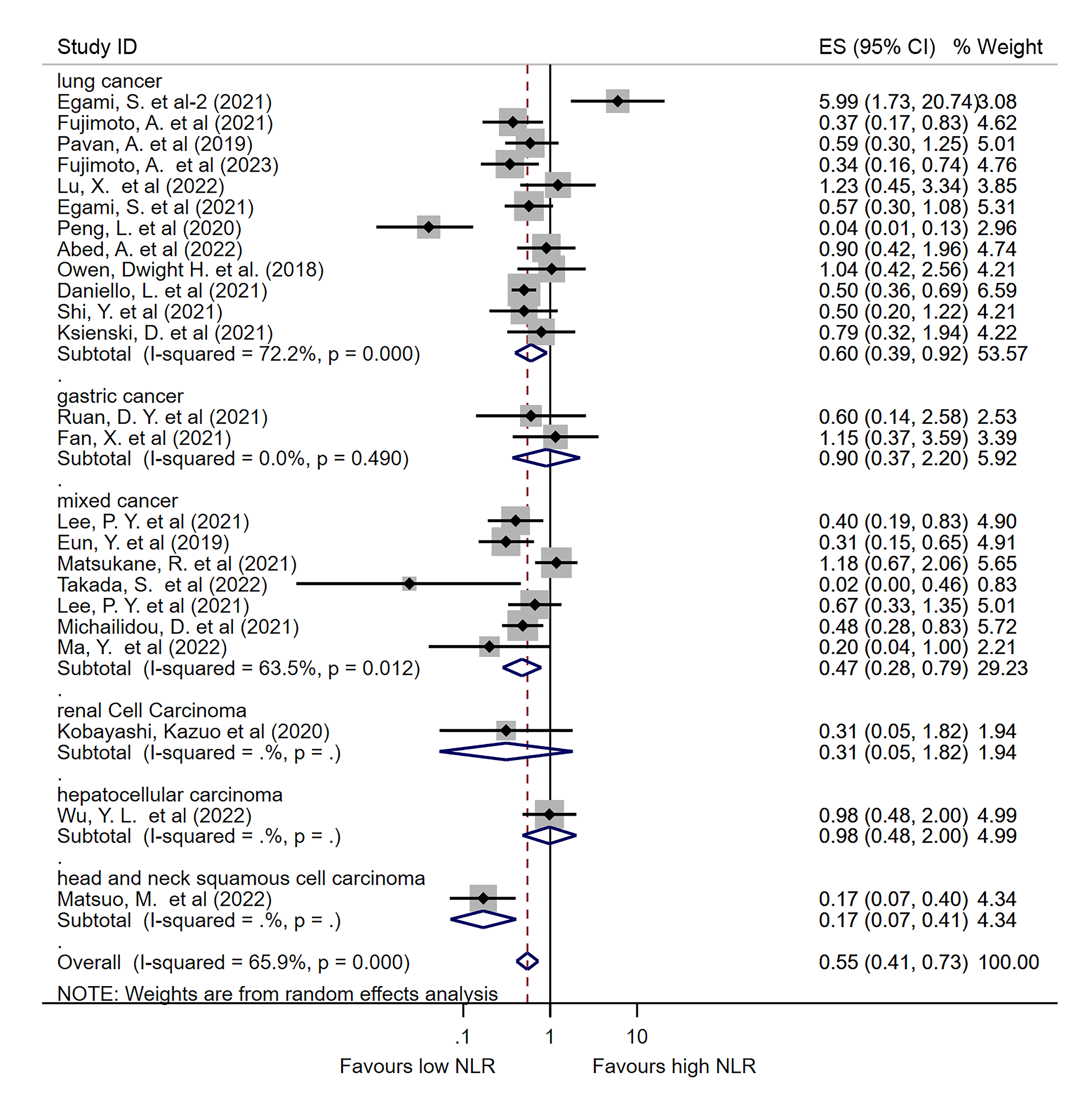

Supplement: Supplementary Figure 4 — Subgroup analysis of the pooled ORs in terms of cancer type. [file Image_4.tif]

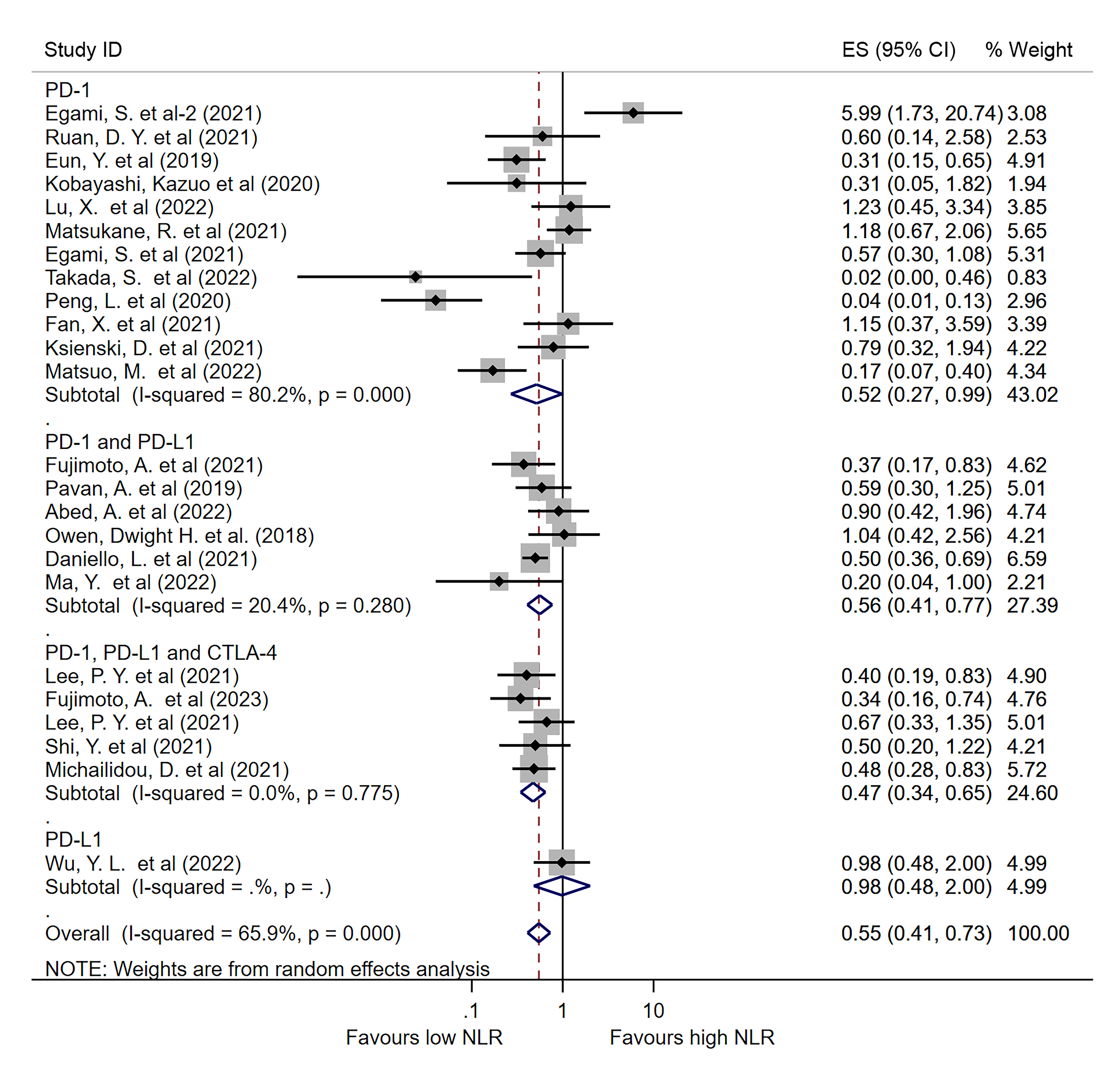

Supplement: Supplementary Figure 5 — Subgroup analysis of the pooled ORs in terms of ICI type. [file Image_5.tif]

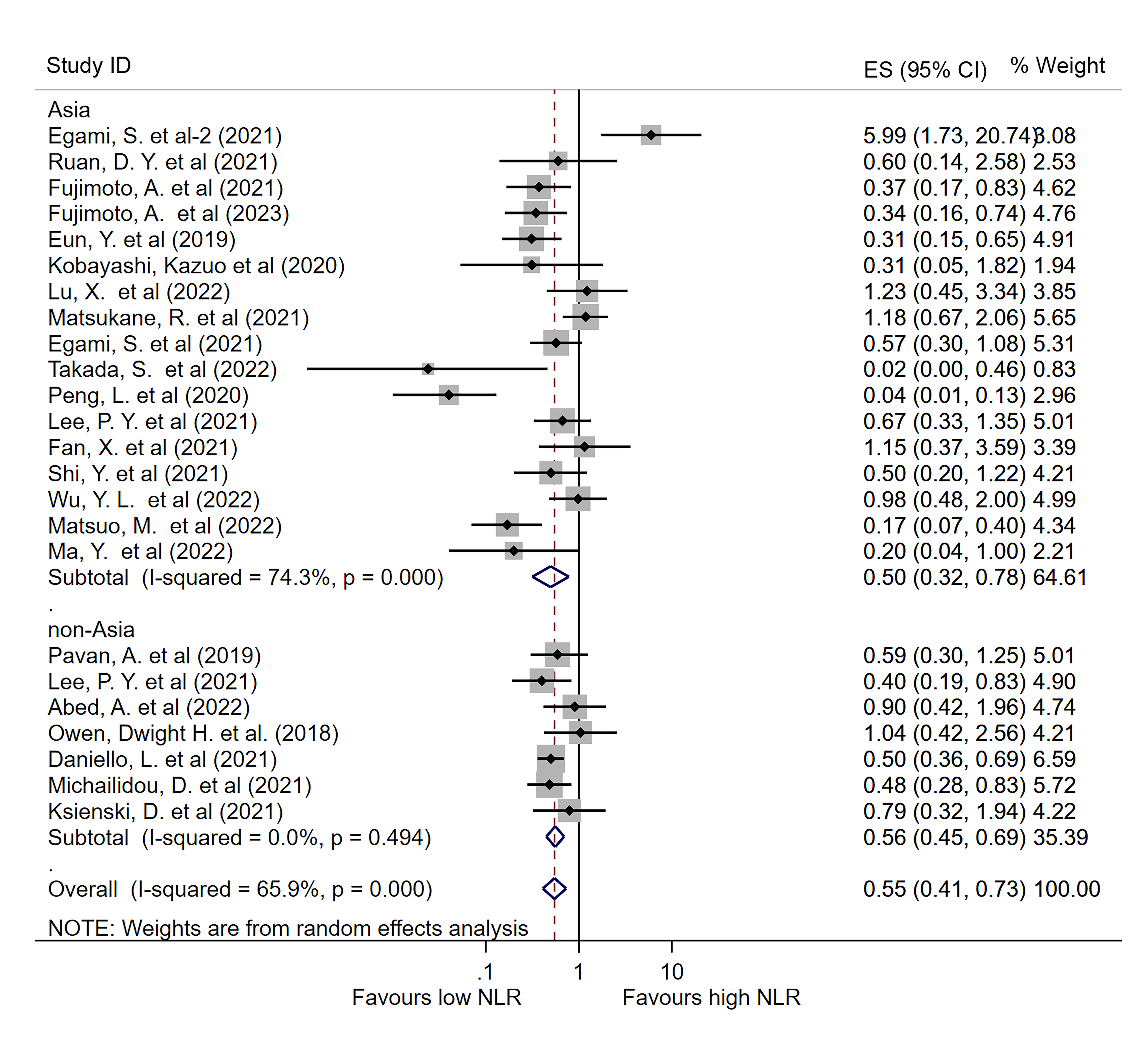

Supplement: Supplementary Figure 6 — Subgroup analysis of the pooled ORs in terms of publication area. [file Image_6.tif]

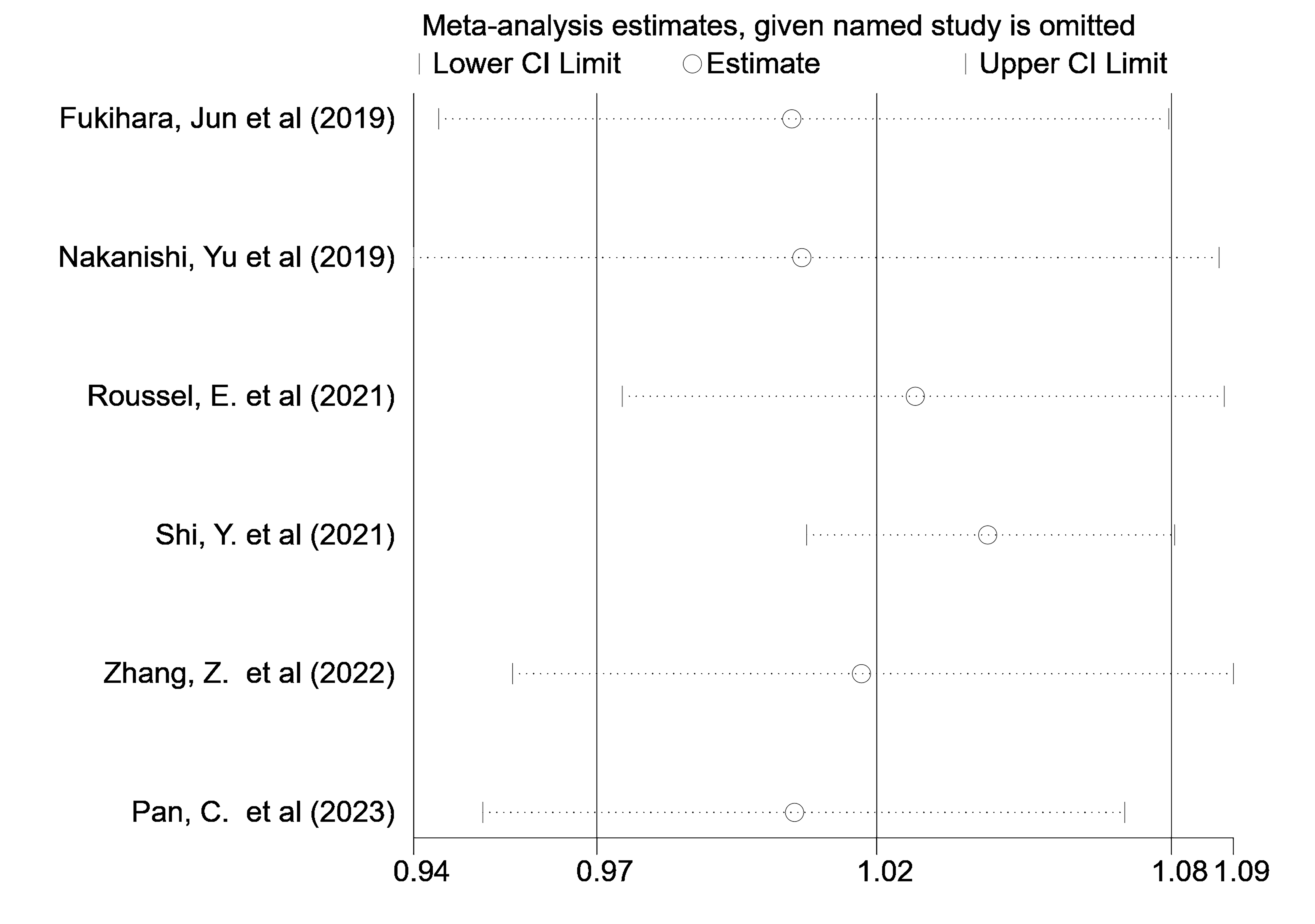

Supplement: Supplementary Figure 7 — Sensitivity analysis of included studies for continuous NLR data. [file Image_7.tif]

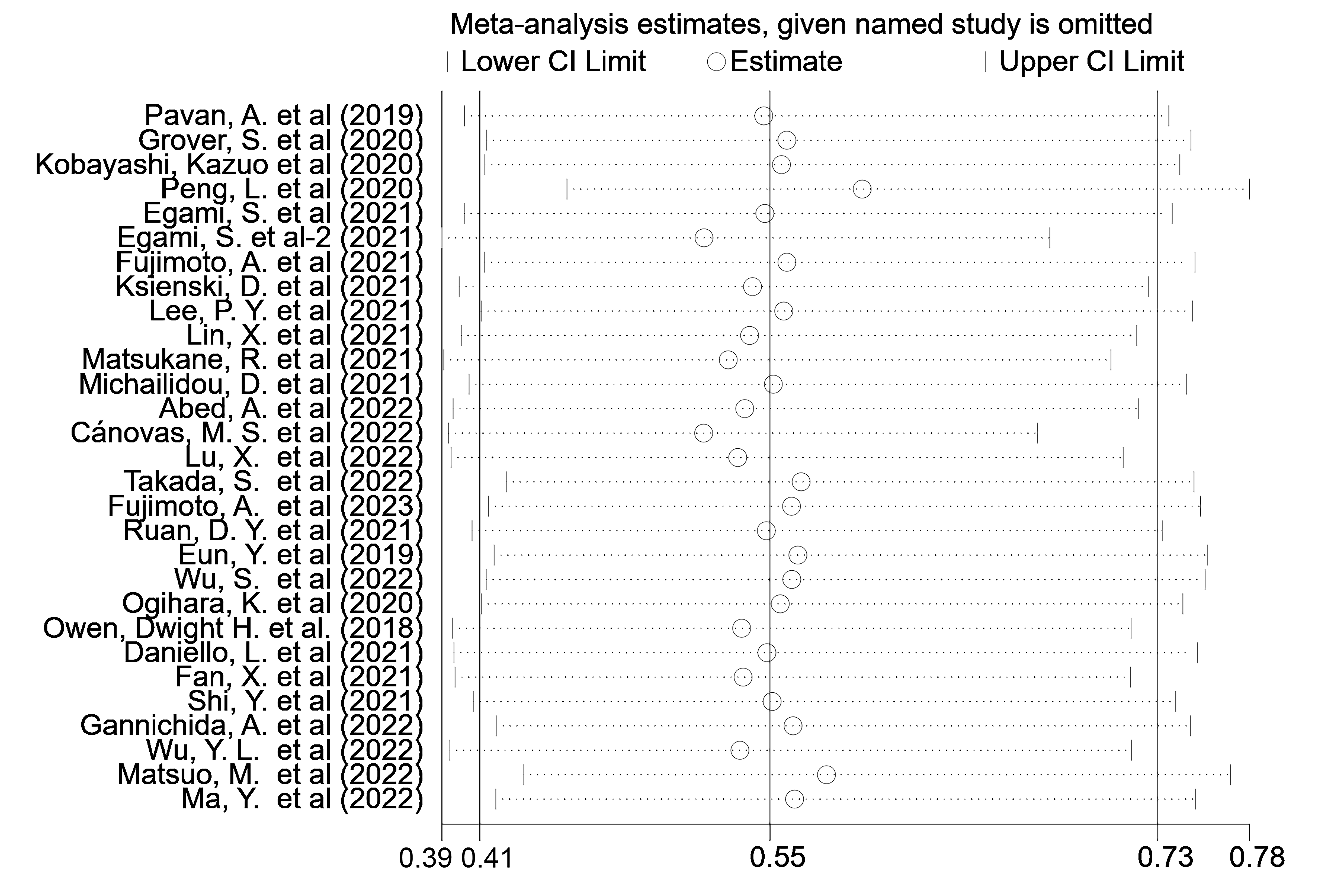

Supplement: Supplementary Figure 8 — Sensitivity analysis of included studies for categorized NLR data. [file Image_8.tif]

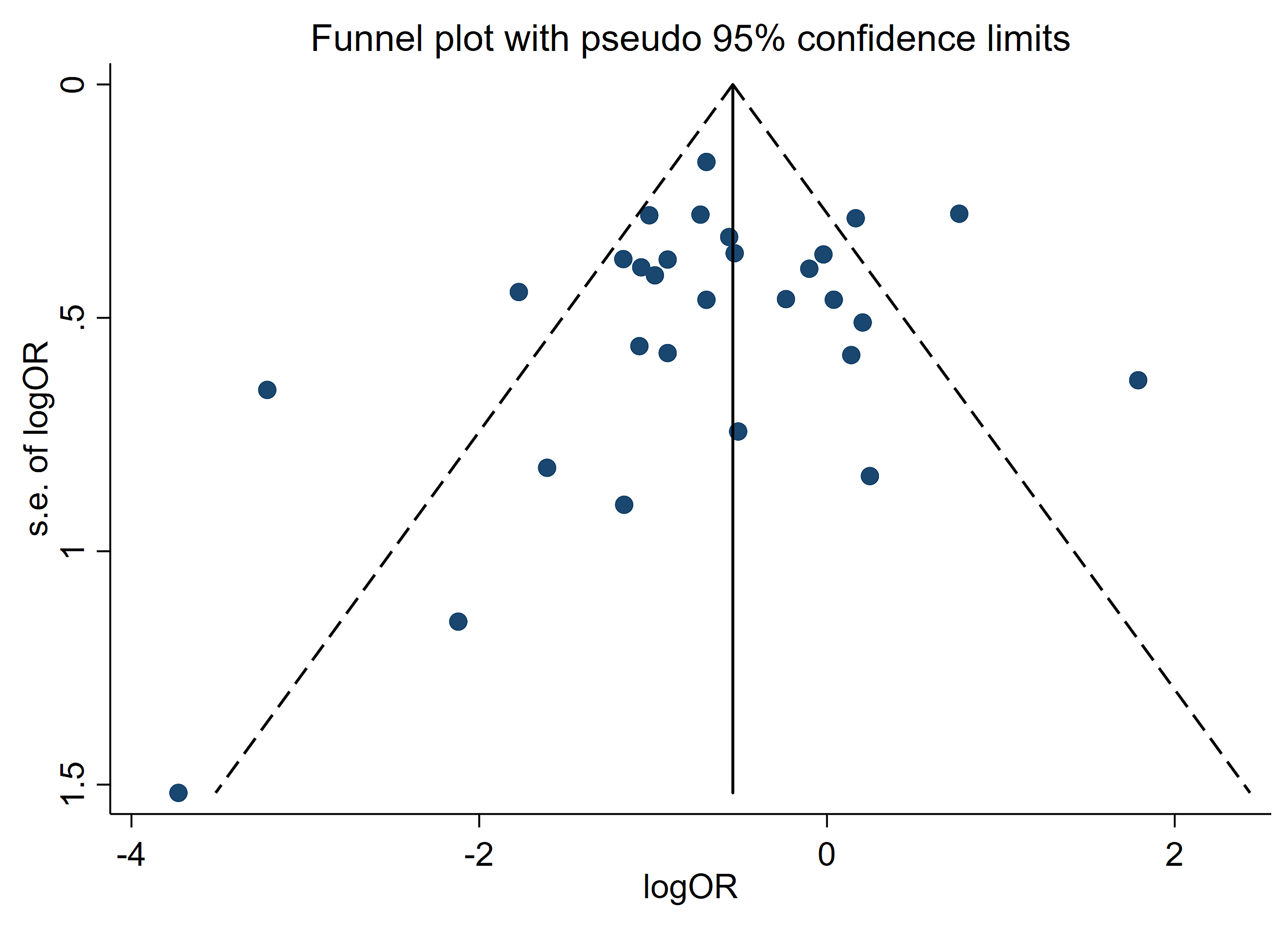

Supplement: Supplementary Figure 9 — Funnel plot showing the publication bias assessment of the included studies. [file Image_9.tif]
